# Refining a steroidogenic model: an analysis of RNA-seq datasets from insect prothoracic glands

**DOI:** 10.1186/s12864-018-4896-2

**Published:** 2018-07-13

**Authors:** Panagiotis Moulos, Alexandros Alexandratos, Ioannis Nellas, Skarlatos G. Dedos

**Affiliations:** 1 HybridStat Predictive Analytics, Aiolou 19, 10551 Athens, Greece; 2 Biomedical Sciences Research Center ‘Alexander Fleming’, Fleming 34, 16672 Vari, Greece; 3 Department of Biology, National and Kapodistrian University of Athens, 15784 Athens, Greece

**Keywords:** Steroidogenesis, Ecdysone, Steroid hormone, Prothoracic gland, Ring gland, *Bombyx mori* genome, Halloween genes, Insect orthology

## Abstract

**Background:**

The prothoracic gland (PG), the principal steroidogenic organ of insects, has been proposed as a model for steroid hormone biosynthesis and regulation.

**Results:**

To validate the robustness of the model, we present an analysis of accumulated transcriptomic data from PGs of two model species, *Drosophila melanogaster* and *Bombyx mori*. We identify that the common core components of the model in both species are encoded by nine genes. Five of these are Halloween genes whose expression differs substantially between the PGs of these species.

**Conclusions:**

We conclude that the PGs can be a model for steroid hormone synthesis and regulation within the context of mitochondrial cholesterol transport and steroid biosynthesis but beyond these core mechanisms, gene expression in insect PGs is too diverse to fit in a context-specific model and should be analysed within a species-specific framework.

**Electronic supplementary material:**

The online version of this article (10.1186/s12864-018-4896-2) contains supplementary material, which is available to authorized users.

## Background

In all animal models studied so far, steroidogenic cells are defined by the expression of cholesterol-metabolizing enzymes to convert it to biologically active steroids [1, 2]. Although vertebrates synthesize and release a variety of steroids depending on their site of synthesis, they are all characterized by the cholesterol side chain cleavage by cytochrome P450 (CYP) CYP11A [1]. On the other hand, ecdysozoa are considered to synthesize, in general, a class of steroids characterized by the presence of the cholesterol side chain [2, 3]. In insects, steroidogenesis results in the synthesis of molecules called ecdysteroids and among insect species there are variations in the exact molecule that is secreted by the prothoracic glands (PGs). The *Drosophila melanogaster* ring glands (*Dm* RGs), a composite organ that comprises three glands, the corpora cardiaca (CC), the corpora allata (CA) and the prothoracic gland (PG) [4], secrete Makisterone A [5] in addition to ecdysone, while other species secrete 3-dehydroecdysone or a mixture of 3-dehydroecdysone and ecdysone as in the case of *Bombyx mori* (*Bm*) [6]. Irrespective of the secreted end product, there exists a core component of ecdysteroidogenesis-regulating enzymes in insects that have been termed Halloween genes [7] and numerous studies have clarified their critical role in ecdysteroidogenesis [2].

Beyond the well-established role of vertebrate steroidogenic enzymes described extensively in authoritative reviews [1, 8–10], research on mammalian cells has identified a large complex of proteins, termed the transduceosome [10–12], which mediates the transport of cholesterol across the outer mitochondrial membrane (OMM) to the inner mitochondrial membrane (IMM). This enables the conversion of cholesterol to pregnenolone, the precursor of all mammalian steroids, by the mitochondrial CYP11A1 [13], which is the rate-limiting step in steroidogenesis. The activity of this large multi-protein complex is regulated by signalling molecules such as the MAPK3/ERK1/2, which phosphorylates and activates StAR [10, 14–16] and the cAMP-dependent protein kinase-regulatory subunit Iα (PKA-RIα), which also mediates phosphorylation and activation of StAR [11].

While in mammalian cells the transport of cholesterol across the OMM to the IMM is the rate-limiting step in steroidogenesis [1] and the step most acutely influenced by second messengers [9], in insects the rate-limiting step of steroidogenesis has been termed the “Black Box” [2, 7], the reaction between 7-dehydrocholesterol (7dC) and D^4^-diketol considered to be taking place before the transport of 2,22-dideoxyecdysone to the mitochondria where it is further converted to ecdysone by the Halloween enzymes, Disembodied and Shadow [2, 7, 17]. Thus, the order of biosynthetic reactions that lead to steroid synthesis is different between insects and mammals: In insects, conversion of cholesterol to ecdysone is considered to begin outside the mitochondria by the microsome-localized [2, 17, 18] protein termed Neverland, which converts cholesterol to 7dC [19]. This step is followed by the biosynthetic reactions taking place within the “Black box” considered but not proven to be mediated by two other enzymes, Spook and Shroud [2, 7, 17]. All research evidence [2, 7, 17] show that the subsequent biochemical conversions lead to the generation of 2,22-dideoxyecdysone, which is transported to the mitochondria only after the conversion of 2,22,25-trideoxyecdysone to 2,22-dideoxyecdysone [2, 7, 17] by another Halloween enzyme, the microsome-localized Phantom [20]. In mammals, after the rate-limiting step of cholesterol transport from the OMM to the IMM is accomplished [10], the first modification of cholesterol takes place by the action of CYP11A1 [1, 13], followed by the efflux of pregnenolone out of the mitochondria to be further modified by other CYP enzymes [1]. Efflux of the mitochondrial steroid product pregnenolone to the endoplasmic reticulum (ER) is thought to occur by free diffusion [8] while influx of the steroidogenesis substrate, cholesterol, to the mitochondria has been shown to occur through mitochondria-associated membranes (MAMs) where the protein ATAD3 is a critical component of the process [21]. Although the mechanism of ecdysone efflux from the mitochondria in PGs is unknown, research on *Dm* RGs has shown that secretion of steroid hormones such as ecdysone may not be simply taking place by diffusion across the plasma membrane, but may be actively released from steroidogenic cells by a mechanism that involves calcium signalling and an ABC transporter termed Atet, which fills secretory vesicles with steroid hormone [22].

While the order of steroid biosynthesis reactions appears to be different between mammals and insects, the mechanism of intracellular cholesterol trafficking that takes place in the early steroidogenic steps [8] appears to be mechanistically similar in steroidogenic mammalian and insect cells. One well-documented common feature is the involvement of Niemann-Pick C1 protein in cholesterol trafficking from late lysosomes to the ER in both mammalian and insect cells [8, 23–25]. To sum up the current knowledge of the steroidogenic cascade, in PG cells [2, 26] suitable sterols are first delivered to the ER to be converted to 2,22-dideoxyecdysone, then transported to the mitochondria to be converted to ecdysone which then diffuses from the mitochondria to be actively released by the action of Atet [22]. In mammalian cells, cholesterol moves from the late lysosomes to the ER [8, 10] and then to the mitochondria through the action of the transduceosome [13]. It is then converted to pregnenolone, which diffuses to the ER to be converted to tissue-specific steroid(s) whose precise mode of secretion is currently unknown [22].

Several studies have made available transcriptomic data from RGs of model insect species taking advantage of developments in transcript analysis and the powerful genetic model of *Drosophila melanogaster* [4, 22, 27–32]. While each study highlights a different set of regulatory mechanisms, the wealth of all this available data poses one important question: Are patterns of steroidogenic gene expression in the insect PGs so highly conserved to justify it being proposed as a model for steroid hormone biosynthesis and regulation [4]? In other words, is there a universal cascade in insect steroidogenic cells from cholesterol import to steroid secretion or is the steroidogenic process species-specific and not context-specific?

To answer these questions and draw conclusions on what universally applies among the various studies in insects, we carried out an analysis of six datasets from two model insects, *Drosophila melanogaster (Dm)* and *Bombyx mori (Bm)*, encompassing eight different studies that also include phenotypic data from RG-specific gene silencing by RNAi [4, 22, 27–32]. Our aim was to refine this steroidogenic model, the PGs, by identifying which genes are commonly expressed in the PGs of the two model insect species. To be able to compare RNA-seq data from *Dm* RGs [31] with data from *Bm* PGs, we re-analysed our RNA-seq data from *Bm* [27, 28] using Cufflinks [33]. We also re-analysed another *Bm* RNA-seq dataset [29, 30] and consolidated the datasets with microarray data that identified *Dm* RG-specific transcripts [4]. These datasets were then enriched with data from prothoracicotropic hormone (PTTH)-stimulated PGs from *Bm* and *Dm* [4] and data that identified the resulting phenotype of RG-specific gene knockdown, an amazing feat made possible only in *Drosophila melanogaster* [32]. We find that beyond a small subset of genes that are shared by the two species, there is very little in common in the signalling mechanisms and expression patterns of Halloween genes that drive ecdysteroidogenesis between these species. On the other hand, we find that the components of the transduceosome that has been identified in mammalian cells [10, 13, 16, 34] are present in PG cells of both species. The PGs can be viewed as a steroidogenic model within a refined framework that includes the transduceosome regulatory cascade, the steroid biosynthesis reactions and transporter-mediated cholesterol and steroid trafficking. Our analysis does not support the existence of highly similar gene regulatory networks and signalling cascades between *Drosophila melanogaster* and *Bombyx mori* which should be analysed in a species-specific context.

## Results

### Analysis of RNA-seq data from the prothoracic gland of *Bombyx mori* and the ring gland of *Drosophila melanogaster*

We analysed our previously published RNA-seq data (SRA Acc. No. SRP062258) [27] *de novo* using Cufflinks [33] to draw comparisons with RNA-seq data from *Dm* RGs [31]. Our *Bm* RNA-seq data from V-0 and V-6 of the 5th instar match the available *Dm* RNA-seq data [31] and provides insights on the ecdysteroidogenic mechanisms between these two developmental time points. On V-0, the high juvenile hormone titre as well as the neurally-derived Bommo-FMRFamide (BRFa) suppress the ecdysteroidogenic activity of the PGs [35, 36], whereas on V-6, which is the day of the onset of wandering behaviour in *Bm*, the juvenile hormone titre is low, PG cells secrete high amounts of ecdysteroids [35, 36] and the PG cells are not fully stimulated by prothoracicotropic hormone [36].

**Fig. 1:**
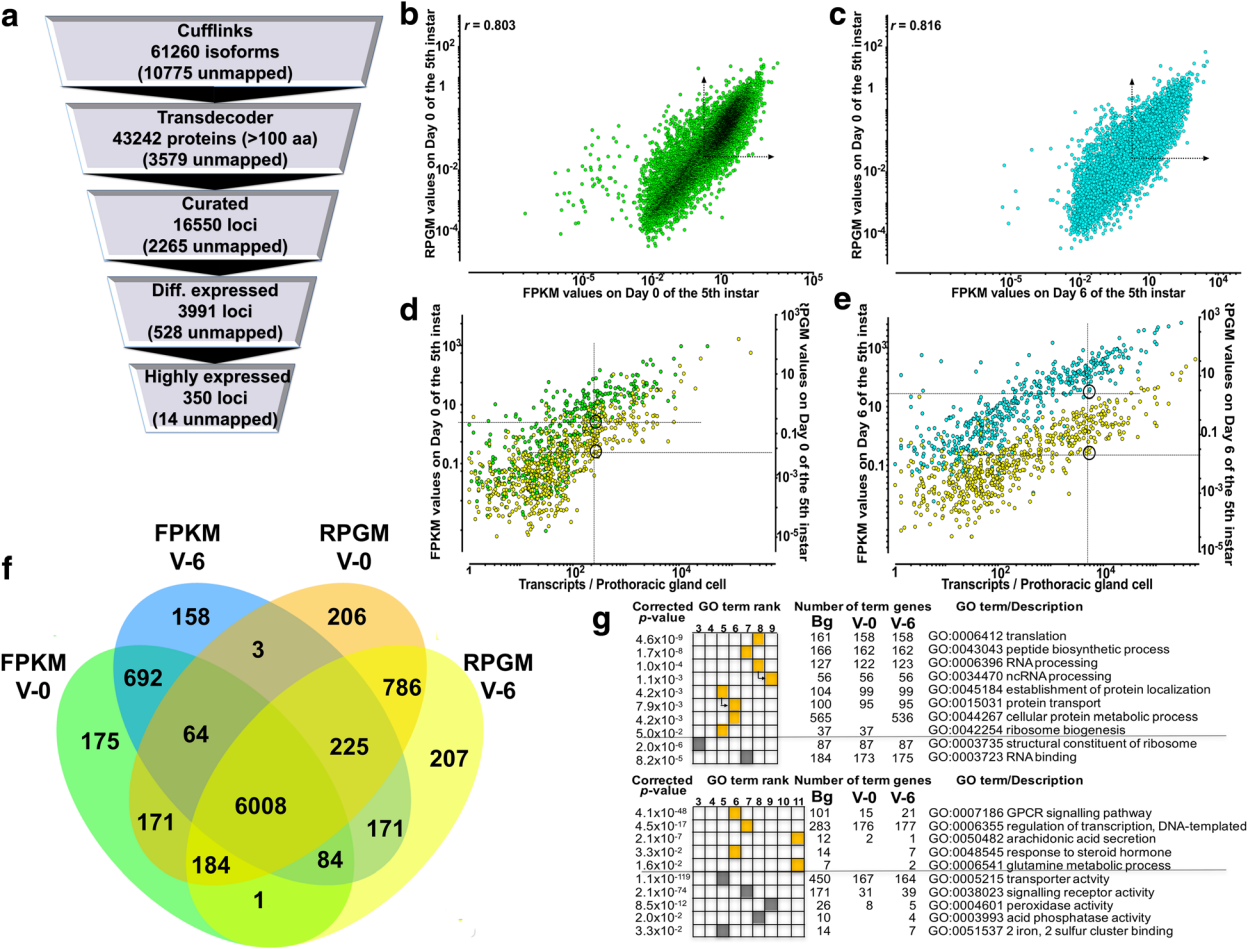
Quantitative and qualitative parameters of gene expression in *Bombyx mori* prothoracic glands. **a**: Schematic workflow that identified genes differentially (Diff.) and/or highly expressed in the *Bm* PGs on V-0 and V-6 of the 5th instar. **b**: Correlation between the Bowtie2 and Cufflinks results output on V-0 of the 5th instar. For each gene, the RPGM (Reads Per Gene Model) derived from Bowtie2 were plotted against the FPKM (Fragments Per Kilobase of transcript per Million of mapped reads) derived from Cufflinks. Dashed arrows define the coordinates above which all genes are considered as expressed (see text for details). **c**: Correlation between the Bowtie2 and Cufflinks results output on V-6 of the 5th instar. For each gene, the RPGM (Reads Per Gene Model) derived from Bowtie2 were plotted against the FPKM (Fragments Per Kilobase of transcript per Million of mapped reads) derived from Cufflinks. Dashed arrows define the coordinates above which all genes are considered as expressed (see text for details). **d**: Determination of the critical RPGM and FPKM value above which all genes are considered as expressed on V-0 of the 5th instar. For each gene, the RPGM values derived from Bowtie2 versus the transcript levels (yellow circles), determined by qPCR, and FPKM values derived from Cufflinks versus the transcript levels (green circles), determined by qPCR, are plotted. Dashed lines indicate the coordinates above which all genes are considered as expressed. Circles indicate the gene with the least RPGM and FPKM value that was identified as expressed by qPCR (see text for details). **e**: Identification of the critical RPGM and FPKM value above which all genes are considered as expressed on V-6 of the 5th instar. For each gene, the RPGM values derived from Bowtie2 versus the transcript levels (yellow circles), determined by qPCR, and FPKM values derived from Cufflinks versus the transcript levels (green circles), determined by qPCR, are plotted. Dashed lines indicate the coordinates above which all genes are considered as expressed. Circles indicate the gene with the least RPGM and FPKM value that was identified as expressed by qPCR (see text for details). **f**: Venn diagram comparing genes considered as expressed in *Bm* PGs on V-0 or V-6 of the 5th instar. Identification of expressed genes was based on FPKM and RPGM values being higher than the minimum estimates from Fig. 1d and e. **g**: g:Cocoa [40] results showing statistically significant overrepresented (upper panel) and underrepresented (lower panel) GO terms assigned to genes expressed on V-0 or V-6 of the 5th instar. Yellow squares show biological processes and dark green squares show molecular functions. Arrows indicate the immediate hierarchical relationship of each term

The Cufflinks output (Additional file 1: Table S1) was supplied to TransDecoder [37] to retrieve the protein sequences encoded by the mapped transcripts (Fig. 1a). The TransDecoder output consisted of 43,242 entries (Additional file 2: Table S2) that were manually curated and filtered down to 16,550 loci encoding non-redundant protein sequences. Of these 16,550 loci, 8914 (53,8%) had 100% similarity with *Bm* Refseq proteins (NCBI annotation 102), 2265 could not be mapped to *Bm* gene models (Fig. 1a) and 1239 had no similarity with the Refseq proteins (Fig. 1a and Additional file 3: Table S3). Moreover, 3991 loci had different expression levels between these two developmental time points while 350 loci showed very high expression levels (> 1,000,000 reads per locus) in either or both of these days (Additional file 3: Table S3). To test for differences in splicing that may affect the expression of genes between V-0 and V-6 we used the *Cuffdiff* parameter of Cufflinks [33] to find 231 loci with differential splicing patterns between V-0 and V-6 (Additional file 4: Table S4) which we further annotated (Additional file 5: Table S5) to remove duplicates and identify 98 unique loci (Additional file 6: Table S6). GO term analysis of the proteins encoded by these 98 loci did not reveal any statistically significant enrichments in either biological process or molecular function.

To assess the quality of the Cufflinks analysis, we plotted the Cufflinks values (Additional file 3: Table S3) against the Bowtie2 values on gene models from our previous analysis [27, 28] (shown also in Additional file 3: Table S3). We find a poor correlation between the two values in both V-0 and V-6 (Fig. 1b and c) an indication that about 20% of the identified *Bm* gene models are either erroneously annotated or do not match with the transcripts provided by Cufflinks. Instead of arbitrarily using the median FPKM value, as we did previously with RPGM values [27], or use an arbitrary value of 1, as done for RNA-seq data from *Dm* RGs [31], above which all genes are considered as expressed, we determined the number of genes that are expressed on V-0 and V-6 by analysing by qPCR the expression of a sample of 650 genes throughout the 5th instar and the first day of pupal stage (Fig. 1d and e). We then plotted the qPCR data (expressed as transcript per prothoracic gland cell) [27] against the FPKM values from Cufflinks and the RPGM values from PANDORA (Fig. 1d and e, see also Additional file 7: Table S7) [38, 39]. The results showed that a value of ≥2.873 FPKM for V-0 and ≥ 3.061 FPKM for V-6 can be used as a safe estimate of gene expression (see Additional file 7: Table S7). These values corresponded to expression levels of *BmBestrophin-4* on V-0 and *BmLMBR1L* [27] on V-6, respectively (Additional file 7: Table S7). Comparing the qPCR results with the RPGM data of these 2 genes from our previous analysis [28], we find that a value of ≥0.0259 RPGM for V-0 and ≥ 0.0253 RPGM for V-6 can be used as a safe estimate of gene expression based on the PANDORA analysis [27] (Additional file 7: Table S7). Plotting these three values (i.e. FPKM, RPGM and transcripts per cell) for each gene confirmed our previous assumption that a large number of gene models in the KAIKObase database are erroneously annotated (Fig. 1d and e). This observation is numerically illustrated in the Venn diagram (Fig. 1f) which shows that 1025 loci identified by Cufflinks as expressed in PG cells, do not correspond to *Bm* gene models. The Venn diagram shows that there are 6008 genes that can be identified as expressed in *Bm* PGs (Fig. 1f) by both methods of evaluation of gene expression. By plotting in a Venn diagram the identified genes, the differentially expressed and the alternatively spliced genes between V-0 and V-6 in *Bm* PGs, only a minor proportion (0.023%) of differential expression can be attributed to alternative splicing (Additional file 8: Figure S1A), and the same minor proportion (0.027%) is observed when only the expressed genes in V-0 and V-6 are considered (Additional file 8: Figure S1B).

We next used g:Cocoa [40] to discover overrepresented and underrepresented gene clusters according to their gene ontology annotation (Fig. 1g and Additional file 3: Table S3). The results show that genes involved in ribosome biogenesis are expressed on V-0 and genes involved in cellular protein modification are overrepresented on V-6 (Fig. 1g and Additional file 3: Table S3). Another noteworthy observation is that genes involved in steroid hormone signalling and synthesis are underrepresented on V-0 (Fig. 1g) providing further support to our choice of the developmental time point to be examined (i.e. V-0).

**Fig. 2:**
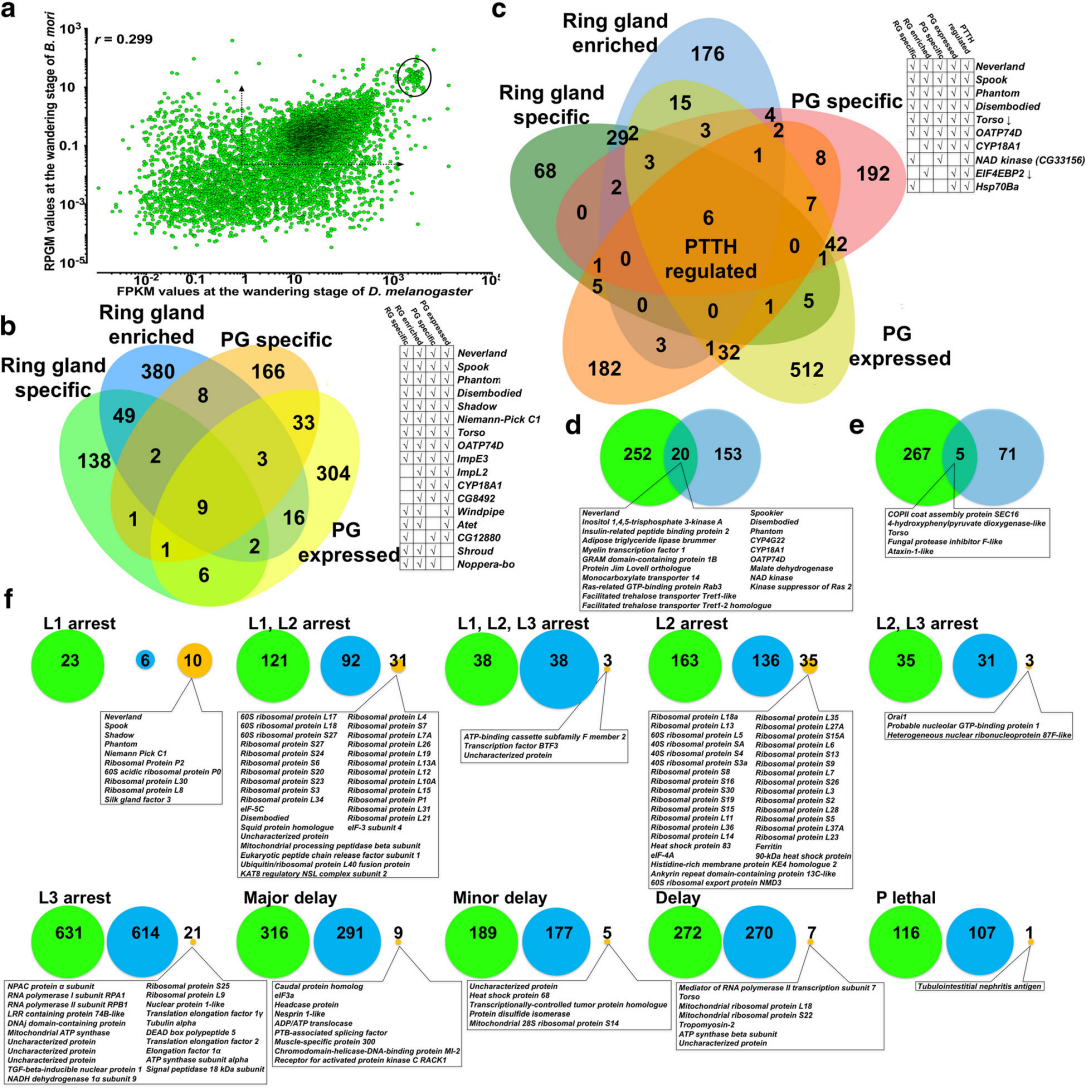
Comparative analysis of gene expression by the prothoracic glands of *Bombyx mori* and *Drosophila melanogaster*. **a**: Correlation between genes expressed by the *Bm* PGs on V-6 of the 5th instar and genes expressed in the *Dm* RGs on the wandering stage as described [31]. For each gene that its orthologue was identified in each of the species, the RPGM (Reads Per Gene Model) derived from Bowtie2 for *Bm* were plotted against the FPKM (Fragments Per Kilobase of transcript per Million of mapped reads) derived from Cufflinks for *Dm* [31]. Dashed arrows define the values above which all genes are considered as expressed in either species [31]. The circled area indicates a cluster of highly expressed ribosomal proteins in both species (see Additional file 12: Table S11). **b**: Venn diagram comparing the *Dm* RG-specific [4] and the *Dm* RG-enriched genes [31] with the *Bm* PG-specific [29, 30] and the *Bm*-PG expressed genes (this study). The adjacent grid highlights the genes found in at least two of these four datasets. **c**: Venn diagram comparing the ring gland-specific [4], the *Dm* RG-enriched [31] genes with the PG-specific [29, 30], the PG expressed genes (this study) and the PTTH-regulated genes [4] of *Bm*. The adjacent grid highlights the genes found in at least three of these five datasets. **d**: Venn diagram comparing the PTTH-upregulated genes in *Bm* PGs [4] with the PG-specific genes [29, 30] (see Additional file 13: Table S12). **e**: Venn diagram comparing the PTTH-downregulated genes in *Bm* PGs [4] with the PG-specific genes [29, 30] (see Additional file 13: Table S12). **f**: Proportional representation of *Bm* and *Dm* orthologous genes whose *Dm* RG-specific silencing resulted in the indicated phenotype as described [32]. Green circles show the number of *Dm* genes whose RG-specific silencing exhibited the indicated phenotype [32], blue circles show the number of their *Bm* orthologues that were not highly expressed in PGs and the orange circles show the *Bm* orthologues that were highly expressed in PGs (see Additional file 3: Table S3, Additional file 15: Table S14 and Additional file 16: Table S15). The sum of blue and orange circles for each group is the number of *Bm* genes orthologous to the *Dm* genes shown in the green circles (see Additional file 9: Table S8). Circles are proportional to each other for each phenotype described in [32]

So as to include the 350 highly expressed genes (Additional file 3: Table S3) for which FPKM values were above the threshold of Cufflinks, we plotted the RPGM values of our *Bm* PGs V-6 RNA-seq data against the FPKM values of RNA-seq data from *Dm* RGs at the wandering stage [31] to identify the degree of correlation between the two species in a temporally comparable developmental time point. Using the clusters of orthologous genes (Additional file 9: Table S8), the results (Fig. 2a) show that there is little correlation (*r* = 0.299) between the expression of the orthologous genes of the two species, besides a cluster of ribosomal proteins that were highly expressed in both the PG and the RG (Fig. 2a).

To identify common and tissue-specific genes between the two species, we annotated and identified the *Bm* and *Dm* orthologues (Additional file 9: Table S8) and generated 4 distinct uncorrelated datasets: 1) of the 272 PG-specific genes derived from RNA-seq data [29, 30] (Additional file 10: Table S9 and Additional file 11: Table S10 and Additional file 12: Table S11), 2) of the 472 expressed and *Dm* RG-specific genes derived from RNA-seq data [31] (Additional file 12: Table S11), 3) of the 208 *Dm* RG-specific transcripts derived from microarray data [4] (Additional file 12: Table S11) and 4) of the 665 genes whose levels differed by 10-fold from V-0 to V-6 and vice versa (Additional file 12: Table S11). We then plotted the common genes identified in these datasets in a Venn diagram (Fig. 2b) which shows that there are only 9 genes expressed in a PG-specific manner in both species. These are the 5 Halloween genes identified in previous studies [2], the prothoracicotropic hormone receptor, *Torso* [41], the *Niemann-Pick C1* gene encoding the cholesterol transporter NPC1, a gene expressing the organic-anion transporter 74D [42] and the ecdysone-inducible gene E3 (*ImpE3*) [43] (Fig. 2b). A sixth Halloween gene (i.e *Shroud*) [44] had a constantly high < 10-fold difference between V-0 and V-6 of *Bm* (Fig. 2b). Most of the other genes shown in the grid (Fig. 2b) have already been described as having a critical role in ecdysteroidogenesis with *Atet* [22] being the most recently assigned one.

To determine which of the genes present in these 4 datasets are regulated by the PTTH signalling cascade, we used RNA-seq data from PTTH-treated *Bm* PGs [4] which we annotated (Additional file 13: Table S12) to add an additional dataset of *Dm* orthologues of PTTH-regulated genes (Additional file 14: Table S13) [4]. By plotting all 5 datasets together in a Venn diagram (Fig. 2c) we identify that only 4 Halloween genes are regulated by the Torso signalling pathway, in addition to the *Torso* and *OATP74D* genes (Fig. 2c). These results are in complete agreement with previously reported results which showed that the ecdysteroidogenesis-promoting PTTH upregulates the JH-dependent expression of the Halloween genes *Neverland*, *Spook*, *Disembodied* and *Phantom*, but not the JH-independent expression of *Shadow* and *Shroud* [45, 46].Among the other genes, most notable is the expression of a gene encoding for a NAD kinase (CG33156) which is a PG-specific [30] and RG-specific [4] gene but not highly expressed in the PGs and RGs of both species (see Additional file 12: Table S11).

We next asked how many PG-specific genes are up- or down-regulated by PTTH in *Bm* PGs using previously published datasets [4, 29, 30]. To answer this, we annotated and compared the RNA-seq data of PTTH-stimulated PGs [4] with our re-analysed RNA-seq data derived from a previous study [29]. The results (Fig. 2d and e) show that PTTH up-regulates 20 PG-specific genes and down-regulates 5 PG-specific genes while there was no statistically significant gene enrichment in all the up-regulated or down-regulated genes. A similar comparison in *Dm* done by another research group [4] using transgenic flies yielded a set of genes (Additional file 13: Table S12 and Additional file 14: Table S13) that were different from those shown in Fig. 2d and Fig. 2e.

Next, we integrated the phenotypic data based on RG-specific in vivo RNAi screen of 12,504 *Dm* genes [32] with our transcriptomic data based on orthologous genes between the two species. Our analysis (see Additional file 9: Table S8, Additional file 14: Table S13, Additional file 15: Table S14 and Additional file 16: Table S15) for the different phenotypes observed in *Dm* (Fig. 2f) [32] shows that there is a core subset of highly expressed genes of *Bm* whose orthologues in *Dm* are developmentally vital (see Additional file 9: Table S8, Additional file 15: Table S14 and Additional file 16: Table S15). Covering almost 1/3 of the highly-expressed genes in PGs (125 of 350) (Fig. 2f), the number of highly expressed genes of *Bm* which are orthologous to *Dm* genes is proportionally decreasing in less severe phenotypes (Fig. 2f and Additional file 9: Table S8, Additional file 15: Table S14 and Additional file 16: Table S15). In our view, this is an indication that ecdysteroidogenesis diverged significantly between these two species and subtler regulatory mechanisms come into play to shape ecdysteroidogenesis by the PGs as development ensues.

### Component dynamics of critical signalling pathways in the prothoracic gland of *Bombyx mori* and the ring gland of *Drosophila melanogaster*

**Fig. 3:**
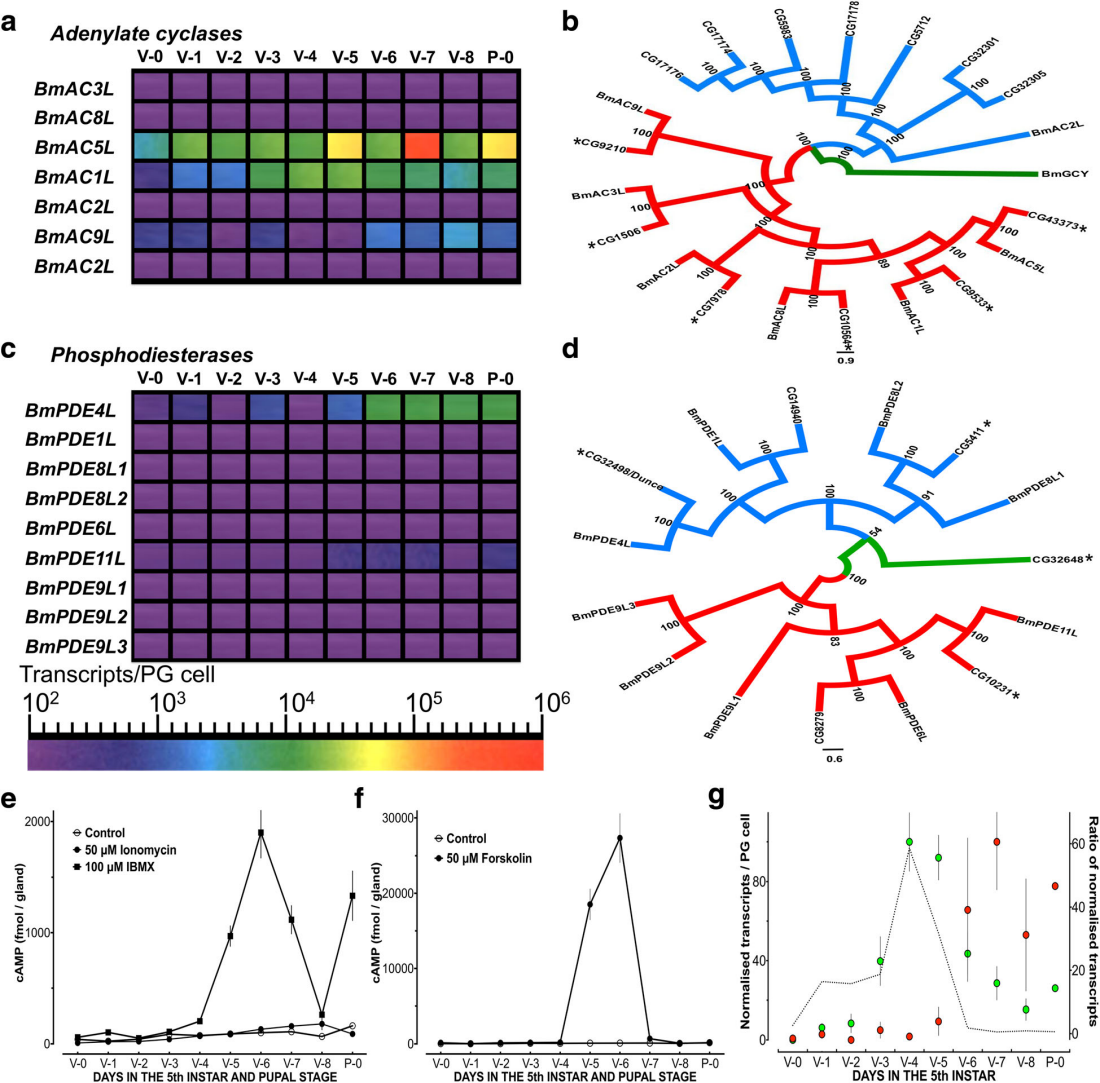
*Bombyx mori* adenylate cyclases and 3′, 5′ cyclic nucleotide phosphodiesterases. **a**: Expression profile heat maps of *Bm* ACs-encoding genes during the final larval instar and the first day of the pupal stage. Abbreviations: V-0 to V-8: Days of the 5th Instar; P-0: First day of the pupal stage. **b**: Posterior probability tree of *Bm* and *Dm* AC proteins. *Bombyx mori* soluble guanylate cyclase (BmGCY) was used as an outgroup. Asterisks indicate the ACs that are expressed in *Dm* RGs based on RNA-seq data [31]. Numbers indicate the posterior probability score (%). **c**: Expression profile heat maps of *Bm* PDE-encoding genes during the final larval instar and the first day of the pupal stage. Abbreviations: V-0 to V-8: Days of the 5th Instar; P-0: First day of the pupal stage. **d**: Posterior probability tree of *Bm* and *Dm* PDEs. *Dm* phosphodiesterase 9 (CG32648) was used as an outgroup. Asterisks indicate the phoshodiesterases that are expressed in *Dm* RGs based on RNA-seq data [31]. Numbers indicate the posterior probability score (%). **e**: Effects of IBMX and Ionomycin on cAMP levels of PGs during the final larval instar and the first day of the pupal stage. Results are expressed as Mean ± SEM, *n* = 4–5. **f**: Effects of forskolin on cAMP levels of PGs during the final larval instar and the first day of the pupal stage. Results are expressed as Mean ± SEM, n = 4–5. **g**: Graphical representation of the cAMP signalling dynamics in *Bm* PGs. Transcript levels of *BmAC1L* (green circles) and *BmPDE4L* (red circles) were normalised against the peak value of each as 100% (V-4 for *BmAC1L* and V-7 for *BmPDE4L*). Dotted line indicates the ratio of normalised *BmAC1L* values/normalised *BmPDE4L* values. Results are expressed as Mean ± SEM, *n* = 4–6

Given the evidence [47] that the cAMP and the IP_3_/DAG signalling pathways play major roles in ecdysteroidogenesis, with the latter reported to be critical for ecdysteroid secretion by *Dm* RGs [22], and the wealth of data on the components of these signalling pathways in *Dm*, the lack of data on the orthologous components in *Bm* presented an obstacle we had to overcome. Thus, we identified the adenylate cyclase, phosphodiesterase, protein kinase C and phospholipase C genes that are present in the *Bm* genome. We then analysed the expression of these families of proteins in the PGs to be able to draw comparisons with the data available for the *Dm* RGs. We identified 7 AC genes present in the *Bm* genome, none of which is correctly annotated (Fig. 3a, Fig. 3b and Additional file 17: Table S16). Multiple sequence alignment of the C1 and C2 domains of the 7 proteins revealed that the critical amino acids for catalytic activity and forskolin binding [48, 49] are conserved in these proteins (Additional file 18: Figure S2A), with the exception of BmAC8L and BmAC5L which possess a Serine to Alanine mutation in their C2 domain (Additional file 18: Figure S2A) that probably renders them irresponsive to forskolin [50]. Domain analysis of the 7 proteins using HMMER shows that they have the characteristic architecture of adenylate cyclases consisting of 2 regions with transmembrane domains interspersed by the two catalytic domains (Additional file 18: Figure S2B). Analysis of expression of adenylate cyclase genes in *Bm* PGs (Fig. 3a) showed that there are 3 AC encoding genes expressed in PGs in very distinct patterns. *BmAC1L*, which is the *Bm* orthologue of *Rutabaga* [51], is strongly expressed in the middle of the 5th instar while *BmAC9L* is expressed mostly in the late 5th instar (Fig. 3a). On the other hand, *BmAC5L*, which was also identified as a highly expressed gene in the PG cells by another group [30] was abundantly expressed in the PG cells with peaks on V-7 and P-0 (Fig. 3a) and it is one of the most abundantly expressed genes (see Additional file 16: Table S15). A posterior probability tree showed that all *Bm* ACs have orthologues in *Dm* with the exception of BmAC2L, the branch of which seems to have expanded considerably in *Dm* (Fig. 3b). Based on RNA-seq data [31], 6 adenylate cyclase genes are expressed in the RGs at the wandering stage of *Dm* but none as high as *BmAC5L* in the *Bm* PGs (Additional file 16: Table S15).

To identify any dynamic interplay between the expression of ACs and PDEs in the PGs, we identified and analysed the expression of PDE encoding genes present in the *Bm* genome. We identified 9 PDE genes present in the *Bm* genome (Fig. 3c). Multiple sequence alignment of their catalytic domains (Additional file 18: Figure S2C) revealed that the critical amino acids for cAMP hydrolysis [52–54] and IBMX binding [55] are highly conserved in *Bm* phosphodiesterases. Domain analysis using HMMER (Additional file 18: Figure S2D) showed that four of the nine proteins are cAMP-specific while the remaining five contain domains and residues similar to cGMP-specific PDEs (Additional file 18: Figure S2C) [52–54]. Analysis of expression of phosphodiesterase genes in *Bm* PGs (Fig. 3c) showed that only one phosphodiesterase gene (*BmPDE4L*), encoding the *Bm* orthologue of *Dunce* [56], is expressed in PGs, while another gene, *BmPDE11L*, had very low expression levels in the late 5th instar. A posterior probability tree showed that most *Bm* PDEs are closely related to their orthologues in *Dm* (Fig. 3d). Based on RNA-seq data [31], 4 PDE encoding genes, among them *Dunce*, are expressed in the *Dm* RGs at the wandering stage (Additional file 16: Table S15).

To resolve the discrepancy between gene expression and ability to be activated by forskolin [50], we measured the cAMP levels of *Bm* PGs upon activation by forskolin, IBMX or ionomycin (Fig. 3e). The results show that forskolin produces massive increases in cAMP levels only on V-5 and V-6 when *BmAC1L* is highly expressed but not on V-7 when *BmAC5L* is highly expressed (Fig. 3f). IBMX produced a 10-fold less increase in cAMP levels on V-5, V-6 and V-7 (Fig. 3e). The pattern of IBMX-induced cAMP increases probably results from the catalytic activity of multiple ACs (Fig. 3a), while ionomycin did not produce any substantial increases in cAMP (Fig. 3e) although it is a potent stimulator of ecdysteroid secretion [57].

We then plotted the expression pattern of *BmPDE4L*, the *Dunce* orthologue, against the expression pattern of *BmAC1L*, the *Rutabaga* orthologue, to identify the cAMP signalling dynamics during the final larval instar of *Bm* (Fig. 3g). In agreement with results from other research groups [58], our results show that the cAMP signalling pathway is dominant in the middle of the 5th instar, until the first major release of PTTH which takes place in the scotophase of V-6 and upregulates the expression of *BmPDE4L* (Additional file 13: Table S12) to suppress cAMP production in the final part of the 5th instar (Fig. 3g).

**Fig. 4:**
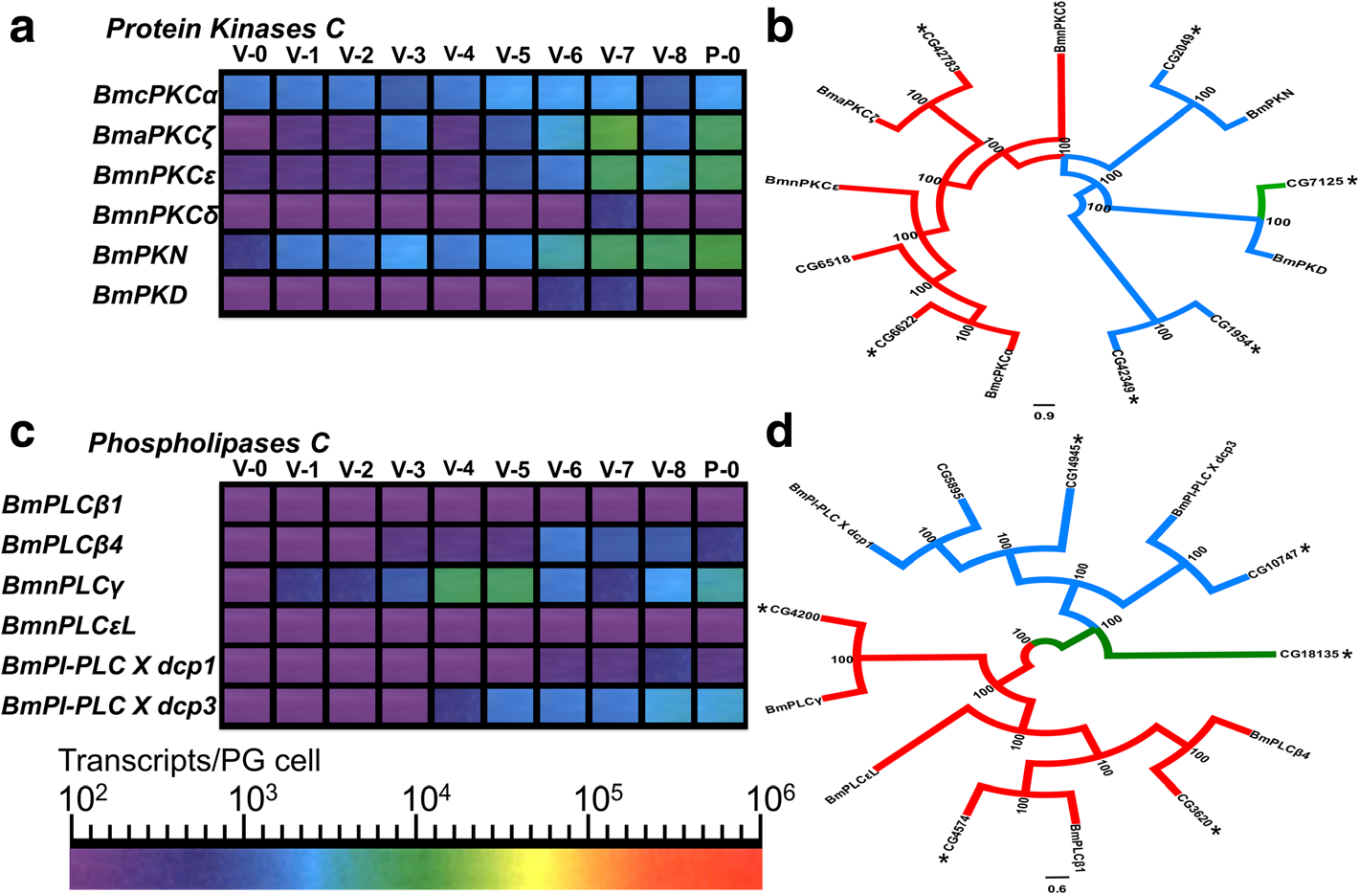
*Bombyx mori* protein kinases C and phosphoinositide phospholipases C (including phosphoinositide phospholipases C X domain containing proteins (PI-PLC X dcp)). **a**: Expression profile heat maps of *Bm* PKCs, PKN and PKD genes during the final larval instar and the first day of the pupal stage. Abbreviations: V-0 to V-8: Days of the 5th Instar; P-0: First day of the pupal stage. **b**: Posterior probability tree of *Bm* and *Dm* PKCs, PKN and PKD proteins. *Dm* PKD (CG7125) was used as an outgroup. Asterisks indicate the PKCs, PKN and PKD proteins that are expressed in *Dm* ring glands based on RNA-seq data [31]. Numbers indicate the posterior probability score (%). **c**: Expression profile heat maps of *Bombyx mori* PLCs and PI-PLC X dcp genes during the final larval instar and the first day of the pupal stage. Abbreviations: V-0 to V-8: Days of the 5th Instar; P-0: First day of the pupal stage. **d**: Posterior probability tree of *Bm* and *Dm* PLCs and PI-PLC X domain-containing proteins. *Dm* glycerophosphodiester phosphodiesterase domain-containing protein (CG18135) was used as an outgroup. Asterisks indicate the PLCs and PI-PLC X dcps that are expressed in *Dm* RGs based on RNA-seq data [31]. Numbers indicate the posterior probability score (%)

Based on previous studies which showed that activation of the ERK signalling pathway lies downstream of the activation of protein kinases C in the PGs of *Manduca sexta* [59–61], we identified and analysed the expression of PKC encoding genes present in the *Bm* genome. We identified 4 PKC genes present in the *Bm* genome, as well as a single gene encoding for a PKN and a single gene encoding for a PKD (Fig. 4a and Fig. 4b). Multiple sequence alignment of the N-terminal phorbol ester and DAG-binding C1 domains [62–64] and the catalytic domain of PKCs [65] revealed that the atypical PKC (*BmaPKCζ*) and *BmPKCδ* probably do not bind phorbol esters and thus do not contribute to the phorbol ester-mediated increase in ecdysteroid secretion by the *Bm* PGs (Additional file 19: Figure S3A and Figure S3B) [66]. The other 2 PKCs possess conserved residues necessary for phorbol ester and DAG-binding (Additional file 19: Figure S3A) [62–64]. Among these two PKC genes, *BmnPKCε* was expressed > 10-fold higher than *BmcPKCα* in the *Bm* PGs while *BmPKCδ* and *BmPKD* had barely detectable levels of expression (Fig. 4a). Three PKC encoding genes (*BmcPKCα*, *BmaPKCζ* and *BmnPKCε*) and *PKN* had peaks of expression on V-7 (Fig. 4a) the day that Bm PGs are stimulated by PTTH [36]. A posterior probability tree showed that *Bm* PKCs considerably diverged from *Dm* PKCs (Fig. 4b). Based on RNA-seq data [31], all but the eye-specific PKC gene, *inaC* (CG6518), are expressed in *Dm* RGs but none, apart from *PKD*, gives an observable phenotype in RG-specific RNAi experiments [32] (Additional file 16: Table S15).

Finally, we identified and analysed the expression of PLC encoding genes present in the *Bm* genome to be able to compare gene expression patterns for this family of genes since proteins encoded by these genes have been shown to mediate ecdysteroidogenesis and ecdysteroid secretion [22, 60, 61, 66]. We identified 2 PI-PLC X domain containing-protein (dcp) and 4 PLC genes present in the *Bm* genome (Fig. 4c). Multiple sequence alignment of the catalytic domain of the 4 PLCs revealed that they all have the residues critical for catalytic activity [67, 68] while PLCβ1 and PLCβ4 contain domains necessary for interaction with Gαq [69] (Fig. 4c — and Additional file 19: Figure S3C and Figure S3D). However, only *PLCβ4* is expressed in *Bm* PGs (Fig. 4c) in contrast with the *Dm* RGs where both *PLCβ1* and *PLCβ4* are expressed [22, 31]. Knockdown of *PLCβ1* in RGs results in major developmental delay [32] (Additional file 17: Table S16). It is noteworthy that *BmPLCγ* shows an unusual expression pattern in *Bm* PGs with high expression on V-4 and V-5 and a drop in its expression on V-7 (Fig. 4c). A posterior probability tree of *Bm* and *Dm* PLCs and PI-PLC X dcps (Fig. 4d) shows that, apart from 2 exceptions, there is almost a one-to-one orthology between the PLCs of both species. Based on RNA-seq data [31], all but one of the genes encoding for a PLC or a PI-PLC X dcp are expressed in *Dm* RGs (Additional file 17: Table S16).

### Expression profiles of cytochrome P450 and ecdysteroidogenesis-regulating genes in the prothoracic glands of *Bombyx mori*

Next, we focused on the CYP family of proteins to benchmark under a single method of absolute quantification the large number of CYP genes identified in publications from different research groups, reviewed in [2, 26], as playing a role in ecdysteroid synthesis and secretion in *Dm* and *Bm*. To accomplish this, we re-analysed and annotated datasets of CYP proteins [70, 71] and integrated them with data from the transcriptome datasets (see Additional file 20: Table S17). A posterior probability tree of *Bm* CYP proteins (Additional file 21: Figure S4) was congruent with previous analysis resolving 4 major clans of CYP proteins [70]. Three of the proteins belonging to the mitochondrian clan (Mito. clan) are encoded by Halloween genes known as *Shadow(CYP315A1)*, *Disembodied(CYP302A1)* and *Shade(CYP314A1)* and two other proteins belonging to the CYP2 clan [70] are encoded by Halloween genes known as *Spook (CYP307A1)* and *Phantom(CYP306A1)* [2, 26].

We analysed the expression of all 82 genes in *Bm* PGs throughout the 5th instar and the first day of the pupal stage and we also analysed the expression of 15 other genes all known or implicated in ecdysteroidogenesis or ecdysteroid metabolism in peripheral tissues.

**Fig. 5:**
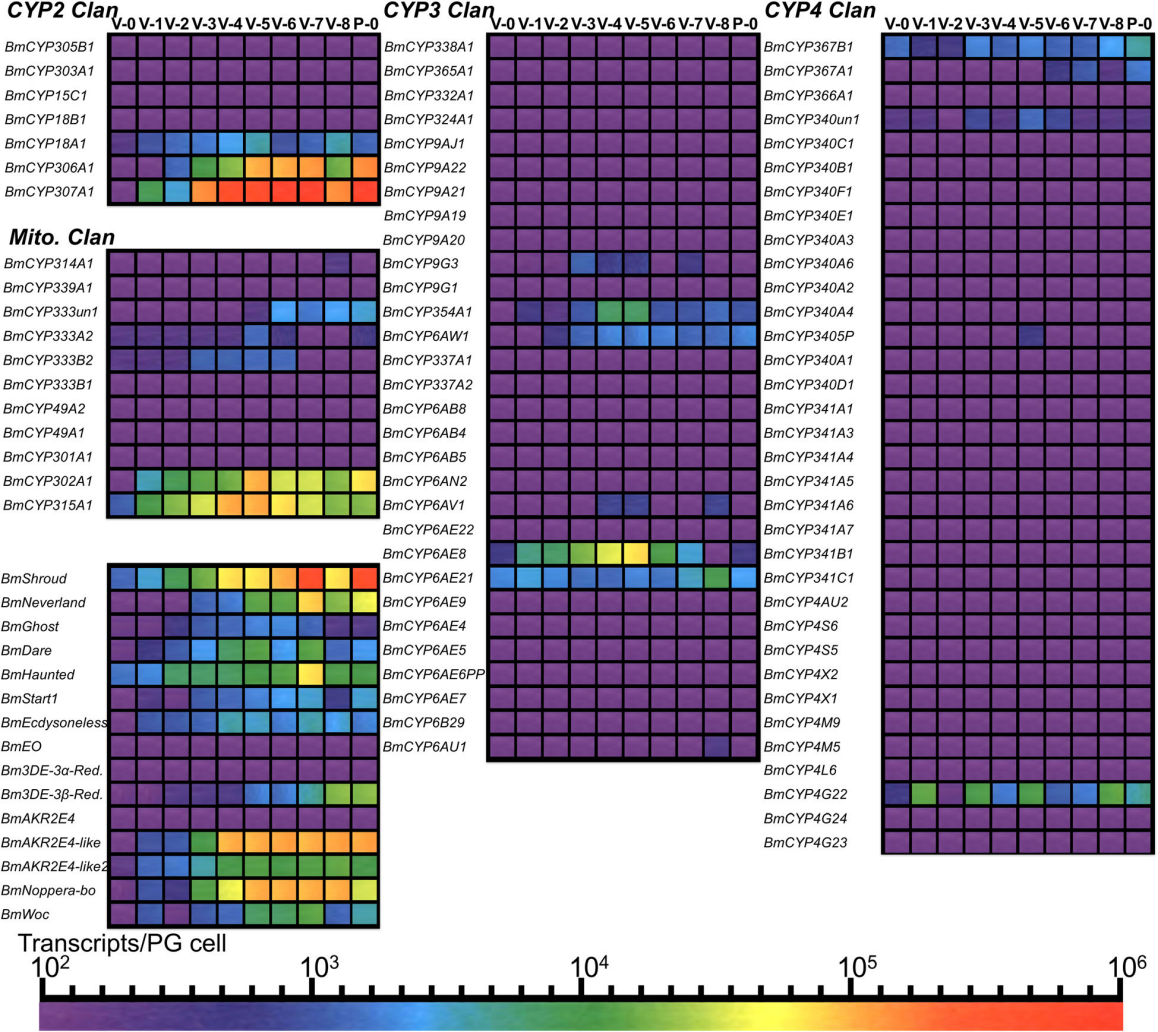
Expression profile heat maps of 82 CYP genes and 15 ecdysteroidogenesis-related genes during the final larval instar and the first day of the pupal stage. Abbreviations: V-0 to V-8: Days of the 5th Instar; P-0: First day of the pupal stage; *Bm*: *Bombyx mori*; EO: Ecdysone Oxidase; 3DE-3α-Red.: 3-DehydroEcdysone-3α-Reductase; AKR2E4: Aldo-Keto Reductase 2E4; BmE: *Bombyx mori* Ecdysone. Nomenclature of CYP genes is based on [70]

We find that the genes *CYP332A1* and *CYP324A1* which are the *Bm* orthologues of *Dm* Cyp6t3 [72] are not expressed in *Bm* PGs (Fig. 5), while the expression *CYP4g25* (alternative name for *CYP4G22*) [70, 71], which has been proposed as a critical player of ecdysteroidogenesis in *Bm* PGs [73], is expressed in *Bm* PGs (Fig. 5).

Among the 82 CYP genes, 23 were found to be expressed in PG cells although most of them had peaks of expression on specific days but were barely detectable in most of the other days examined (Fig. 5). Among those expressed, *CYP307A1*, the *Bm* orthologue of *Spook* and *Spookier*, had the highest expression and in specific developmental time points it was the most abundant transcript of all 650 examined genes (see Additional file 7: Table S7). Besides the CYP genes, we examined the expression of the short-chain dehydrogenase/reductase (SDR) gene, *non-molting glossy/shroud* (*BmShroud*) [44], the cholesterol 7,8-dehydrogenase gene, *neverland* [19], the Halloween genes *haunted* and *ghost* [74] which encode for the subunits of the coat protein complex of COPII vesicles, Sec23 and Sec24CD, respectively, the mitochondrial NADPH:adrenodoxin oxidoreductase gene, *Dare* [75], the *Bm* orthologue of *Start1*(*BmStart1*) [76], the pre-mRNA splicing factor gene, *Ecdysoneless (BmEcdysoneless)* [77], the ecdysone oxidase *(BmEO)* and 3-dehydroecdysone-3α-reductase *(Bm3DE-3α-Red.)* genes [78], the 3-dehydroecdysone 3β-reductase (*Bm3DE 3β-Red.)* gene [78, 79], the 3-dehydroecdysone reductase gene, *AKR2E4* [80] and two closely related isoforms (aldo-keto reductase *AKR2E4-like* and *AKR2E4-like2*, (BMgn009799 and BMgn012152, respectively), the glutathione *S*-transferase gene, *Noppera-bo* [81], *Woc* which encodes for 7,8-dehydrogenase modulator zing-finger protein [82] and the *Dm* orthologue of the nuclear receptor HR4 [72].

There were two patterns of expression immediately evident from the transcript expression analysis in *Bm* PGs. Namely, the mitochondrial genes *Disembodied*, *Shadow* and *Dare* had expression peaks on V-5 followed by reduction of their expression levels late in the 5th instar when ecdysteroid secretion is increasing (Fig. 5 and Additional file 22: Figure S5). On the other hand, the microsomal-localized genes *Spookier*, *Shroud*, *Neverland* as well as *BmStart1*, *BmNoppera-bo*, *BmAKR2E4-like* and *BmAKR2E4-like2* (Fig. 5 and Additional file 22: Figure S5) had patterns of expression that were matching the peaks and troughs of ecdysteroid secretion [27, 36]. Contrary to these patterns, *CYP18A1* which encodes for an ecdysteroid 26-hydroxylase, an ecdysone inactivating enzyme [83], had a pattern of steadily high expression in the first half of the 5th instar followed by a decrease after V-6 (Fig. 5 and Additional file 22: Figure S5). Comparing these patterns with those reported for *Dm* [4], we observe that only *Neverland*, *Spookier* and *CYP18A1* had patterns of expression that matched those in *Bm* PGs (compare Fig. 5 and Fig. 3 in [4]). Finally, we investigated the expression in *Bm* PGs of *HR4*, the gene encoding for the nuclear receptor HR4 which has been proposed as a key regulator of PTTH signalling in both *Bm* and *Dm* PGs [4, 72]. Our results (Additional file 23: Figure S6) show that *HR4* is undetectable in *Bm* PGs until V-6, has a peak of expression on V-7 and then falls to undetectable levels on V-8. This result is consistent with the documented upregulation of *HR4* expression by PTTH stimulation of *Bm* PGs [4] after the reported secretion of PTTH on the scotophase between V-6 and V-7 in the hybrid used in this study [36]. Therefore, the peak of *HR4* expression on V-7 is the result of PTTH stimulation of *Bm* PGs but its transcript levels are barely detectabl*e* on the other days of the 5th instar (Additional file 23: Figure S6).

### Data synthesis towards refining the model

**Fig. 6:**
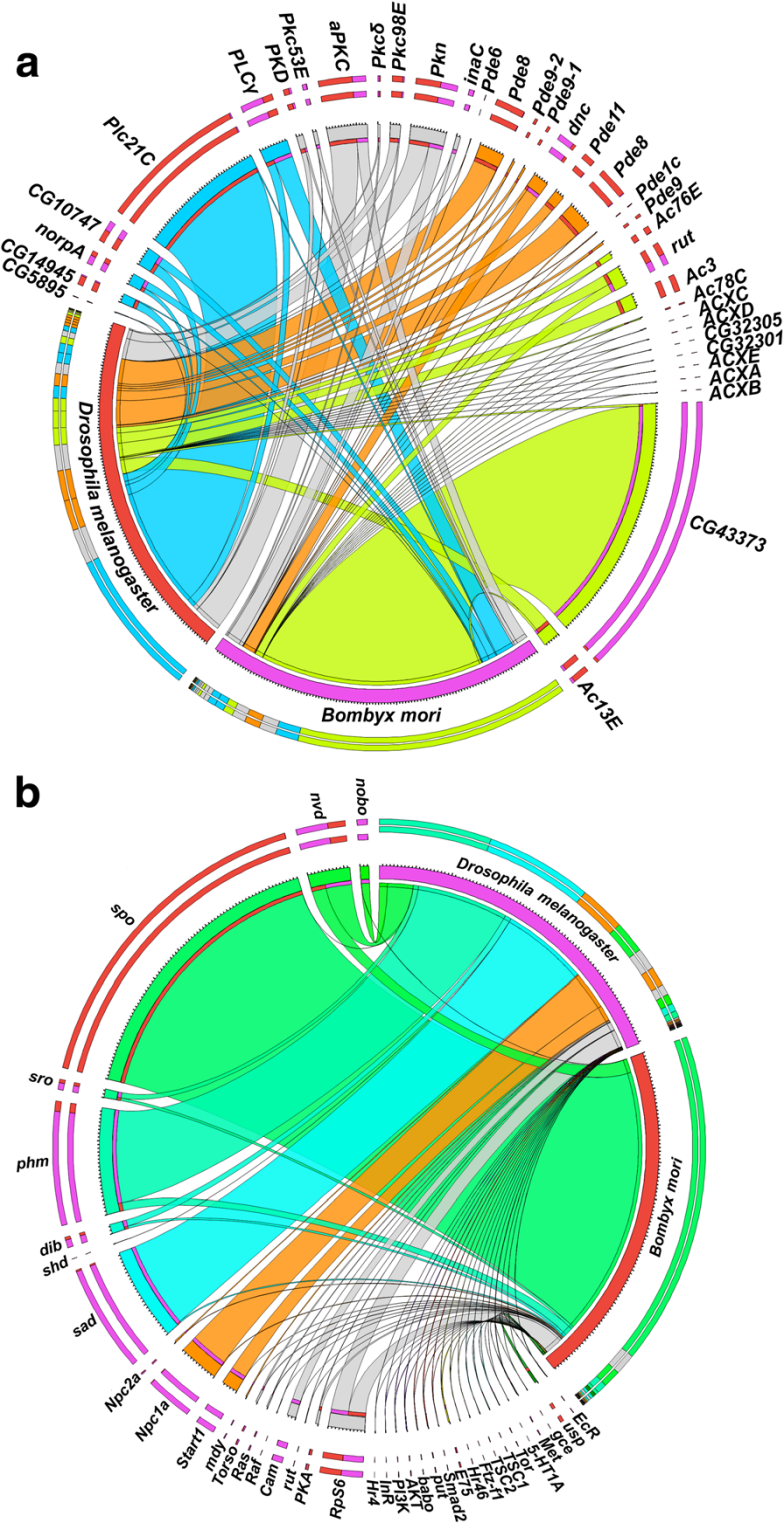
Comparison of expression dynamics of gene groups involved in ecdysteroidogenesis in *Drosophila melanogaster* and *Bombyx mori*. **a**: Circos plot comparing the expression of ACs, PDEs, PKCs and PLCs during the wandering stage of *Dm* [31] and V-6 of the 5th instar of *Bm*. Percentage values are related to FPKM values. See Additional file 17: Table S16 for details. **b**: Circos plot comparing the FPKM values of genes involved in *Dm* ecdysteroidogenesis, as presented in [31], with the RPGM values for the orthologous genes of *Bm*. Percentage values are related to FPKM (*Dm*) and RPGM (*Bm*) values as shown in [28, 31]. See Additional file 9: Table S8 for details

Following the identification of the ACs, PDEs, PKCs and PLCs from *Bm*, we compared the transcript abundance of these genes and their *Dm* orthologues at two comparable developmental time points, i.e. the V-6 of the 5th instar of *Bm* and the wandering stage of *Dm* [31]. The results plotted on a Circos graph [84] (Fig. 6a) show the dominance at the transcript level of *BmAC5L* (orthologue of CG43373) in *Bm* PGs while *Plc21C* (orthologue of *BmPLCβ1*) is the most abundant gene in *Dm* RGs, among those shown in Fig. 6a. The Circos graph shows no congruent pattern of expression of these genes between *Bm* and *Dm*, an indication that the cAMP and IP_3_/DAG signalling cascades in these cells exhibit different dynamic expression patterns between the two species.

We also compared the expression levels of plasma membrane receptors based on our previous results [27] and those available from *Dm* RNA-seq data [31] (see Additional file 24: Table S18) to find that only *Torso* has a PG-specific and PG-enriched mode of expression (Fig. 2 and Additional file 24: Table S18) in both species. There were 4 *Dm* genes encoding for methuselah receptors and a gene encoding for a putative prostaglandin receptor (CG7497), that had > 10-fold enrichment in *Dm* RGs (see Additional file 24: Table S18) [31] while related receptors or orthologues of these genes are expressed in *Bm* PGs [27]. Beyond that, there is very little similarity on the class A and class B GPCR genes that are expressed in the *Bm* PGs and *Dm* RGs on this particular developmental time point of the two species. However, receptors that belong to class D Frizzled/Smoothened group [27] are expressed in both species (Additional file 24: Table S18). The same is true for genes encoding for receptor tyrosine kinases and receptor serine/threonine kinases such as *Egfr*, *Babo*, *wit* and *tkv* which are expressed in PG cells of both species in agreement with previously published results [27, 85]. Our analysis provides no clues to the nature of an unknown GPCR and its cognate ligand implicated as mediators of the calcium signalling event that stimulates ecdysone release from *Dm* RGs [22].

We extended the comparison of PGs from both species by comparing the expression of genes involved in ecdysteroidogenesis as presented for *Dm* RGs [31] with their corresponding orthologues in *Bm* PGs (Fig. 6b). The results show that besides *Neverland*, *Shroud* and *RpS6*, there is very little similarity in relative transcript abundance between the two species (Fig. 6b). Most striking is the difference in the expression of *Spook/Spookier* between the two species (Fig. 6b). Whereas *Spook* is expressed at very low levels in *Dm* RGs, its *Bm* orthologue, *BmSpookier(CYP307A1)*, is the most abundant transcript in *Bm* PGs (Fig. 6b). The most abundant transcript in *Dm* was *Phantom* (Fig. 6b) which also had high levels of expression in *Bm* PGs (Fig. 5).

Finally, we identified the *Bm* and *Dm* orthologues of the multi-protein components of the transduceosome [34] and tabulated their expression patterns in the PGs on V-6 and the wandering stage of *Bm* and *Dm*, respectively. The results (Table 1) show that all the components of the transduceosome are expressed in the PG and RGs of both species in a comparable manner, one notable exception being *START1* which is highly expressed in the RG but lowly expressed in *Bm* PGs (Table 1). By integrating also the proteomic analysis results from our previous study [28], it is evident that the encoding proteins can also be identified, at least, in *Bm* PG cells. As shown in Table 1, knockdown of some of these genes in RGs results in development arrest or delay [32] an indication that the encoding proteins are vital for the timely conclusion of ecdysteroid-mediated larval development.

**Table 1:** Tabulation of expression and phenotypic data of *Drosophila melanogaster* and *Bombyx mori* genes that are orthologous to human genes encoding proteins that comprise the transduceosome [13, 34]

*Bombyx mori* gene ID	Flybase gene ID	FPKM Prothoracic gland	FPKM Ring gland	Phenotype	Orthologue of	Proteome Discoverer®1.4 -assigned score
–	–	–	–	–	*ACOT2/3*	–
BMgn002906	FBgn0031992	325,15	252,53	NOP	*ACBD1(DBI)*	9
BMgn003788	FBgn0263120	43,36	98,55	NOP	*ACSL4*	32.7
BMgn003829	FBgn0003360	0^a^	734,65	major delay	*ANT*	136.2
BMgn003985	FBgn0261014	146,4	524,02	major delay	*ATAD3*	179.5
BMgn004096	FBgn0022382	21,3	43,05	NOP	*PKA-R2*	45.6
BMgn006907	FBgn0001248	280,84	423,08	major delay	*IDH2*	52.5
BMgn007194	FBgn0036892	17,71	36,2	NOP	*LONP1*	68.7
BMgn007586	FBgn0001248	15,67	423,08	major delay	*IDH2*	47.6
BMgn008137	FBgn0011769	10,88	12,93	L3 arrest	*FDX1*	4.7
BMgn008154	FBgn0261276	18,37	45,42	NOP	*OPA1*	34.8
BMgn008523	FBgn0023536	15,08	15,56	NOP	*ACBD3 (PAP7)*	12
BMgn008955	FBgn0004363	0^a^	470,55	NOP	*VDAC*	128.8
BMgn009790	FBgn0035028	6,72	2276,69	NOP	*MLN64/StARD1 (START1)*	–
BMgn011029	FBgn0029969	320,13	101,65	NOP	*ACAT1*	130.1
BMgn011694	FBgn0259243	21,61	33,49	NOP	*PKA-R1*	–
BMgn012659	FBgn0040064	156,94	279,4	NOP	*ACAT1*	115.7
BMgn013063	FBgn0262559	121,72	270,08	NOP	*MDH2*	101
BMgn017207	FBgn0010926	126,01	12,43	L3 arrest	*TSPO*	-^b^
BMgn017400	FBgn0035529	73,26	85,12	L1, L2, L3 arrest	*FDX2*	−^b^
BMgn017553	FBgn0015582	57,92	31,17	L3 arrest	*FDXR (Dare)*	-^b^

Data from the proteomic analysis of V-6 PGs of *Bm* using Proteome Discoverer®1.4 is derived from [28]. Data from phenotypic analysis of RG-specific RNAi-mediated gene knockdown is derived from [32]. See Additional file 9: Table S8 for details

^a^Fragments > 1,000,000

^b^No Uniprot record

## Discussion

In this study we performed an analysis of six different datasets presented in eight different publications to define the framework within which the insect PG cells can be a model for steroid biosynthesis and regulation [4]. To accomplish our goal, we integrated these datasets into a single file (Additional file 12: Table S11) and attempted to identify common regulatory components of the steroidogenic cascade of insect PGs. We extended our search of common regulatory components into vertebrate steroidogenic models to provide a detailed comparison between insect and vertebrate endocrine events at a molecular and cellular signalling level in light of the latest advances in mitochondria cholesterol transport in mammalian steroidogenic cells [1, 8, 10, 13, 34, 86].

Despite the major technical differences between the examined studies, which comprise microarray data [4], RNA-seq data [27–31] and phenotypic data [32], the integrated datasets are in agreement with most, if not all, the published literature on insect steroidogenesis. Although we were initially discouraged by the potential fallibility of the cross-comparison of the datasets, we find that the various datasets are congruent in a species-specific context but less so in a cross-species context.

Using the same criteria as those used in the publications originally describing the datasets, we find that there are few common regulatory components of steroidogenesis conserved between *Bm* and *Dm* (Fig. 2) that can set the PG cells apart from other cells in the insect body. In all 6 datasets examined, only 5 Halloween genes [2], *Torso* [87], *NPC1* [88], *OATP74D* [42, 89], and *ImpE3* [43] (Fig. 2b) met the set criteria, while several genes failed to meet one of the criteria set for each dataset (Fig. 2b). The latter group contains *Atet* [22] that is not specifically expressed in *Bm* PGs, *CYP18A1* [83] that is not specifically expressed in *Dm* RGs and *Shroud* and *Noppera-bo* that showed a steadily high level of expression in *Bm* PGs (i.e not > 10-fold change between V-0 and V-6). It should be noted that *NPC1* [88], *OATP74D* [42, 89] and *ImpE3* [43] have been reported to be expressed in other insect tissues as well, so the results presented here show that they are highly, but not exclusively, expressed in the PGs relative to other tissues. Although the role of several of the genes presented in Fig. 2b and c has been well-documented in insect steroidogenic cells [2, 7, 30, 41, 88], we identified several genes whose role in ecdysteroidogenesis should be further explored (Fig. 2). One of these is the *OATP74D* gene which encodes for a protein with cytoplasmic localisation when overexpressed in a heterologous system [42] but its PG-specific expression in both species merits further investigation. Another example is *CYP18A1*, a gene encoding for a CYP enzyme with 26-hydroxylase activity [83] involved in ecdysteroid inactivation [90], whose enriched expression in *Bm* PGs (Fig. 5) when ecdysone secretion is low [36] indicates that there may exist an interplay between ecdysteroid synthesis and catabolism within the PG cells that dictates the rate of ecdysone release.

One striking difference in the levels of expression of the Halloween genes involved in ecdysteroid synthesis in both species is evident when comparing the levels of expression of *Spook/Spookier* (Fig. 6). The role of this enzyme in ecdysteroidogenesis remains unknown [2], but the very low levels of its expression in *Dm* RGs indicate that this may be the “rate-limiting” step in ecdysteroidogenesis in this species. On the contrary, *Spookier*, the *Bm* orthologue of *Spook* [91], is the most abundant transcript in *Bm* PGs (Fig. 6b), an indication that it may not be the “rate-limiting” step in ecdysteroidogenesis in *Bm* PGs. Its placement in the “Black box” of the ecdysteroidogenic cascade in insects [2] may represent its unknown enzymatic role in ecdysteroidogenesis but should not represent its rate-limiting role in the process. In comparison with other Halloween enzymes, there are more examples of post-transcriptional [92, 93] and post-translational [87] regulation of *Spook* expression and this suggests that this enzyme is under a dynamic regulatory mechanism that may be different in lepidopteran species [87] than in *Drosophila melanogaster*.

Τhe cAMP signalling pathway was the first cellular signalling mechanism that was associated with ecdysteroidogenesis [58] but the importance of this signalling pathway in ecdysteroidogenesis receives less attention ever since the ERK-mediated signal transduction mechanism of PTTH was identified [41]. Recent phenotypic data from RG-specific RNAi-mediated gene knockdown [22, 32] shows that the IP_3_/DAG signalling pathway is the dominant signalling pathway in *Dm* PGs since RNAi-mediated gene knockdown of the cAMP signalling pathway in *Dm* PGs gives no observed phenotype [32]. Conversely, a large number of publications have shown that the cAMP signalling pathway is the prominent mechanism of ecdysteroidogenesis in lepidopteran species [7]. Our results further show that the cAMP signalling pathway is most critical in the middle of the 5th instar of *Bm*, as judged by the sharp increase in expression of *BmAC1L* (Fig. 3a), the *Bm* orthologue of *Rutabaga*, in concert with the low levels of expression of *BmPDE4L* (Fig. 3b), the *Bm* orthologue of *Dunce*, the only phosphodiesterase expressed in *Bm* PGs. However, the very high levels of expression of *BmAC5L* (Fig. 3a) [30] present a puzzle that may be difficult to resolve regarding the role of ACs in PGs and at the same time highlight the inconsistent pattern of gene expression emerging from the comparison between the two species.

This pattern is even more inconsistent between the two species when it comes to transcription factors expressed in the PGs [4] (Additional file 12: Table S11). Of the 13 DNA-binding proteins identified as specifically expressed in the *Dm* RGs [4], only 2 of their orthologues, *timeless* (*tim*) and *vvl* are expressed in *Bm* PGs (Additional file 3: Table S3) and only 1 of these (*timeless* (t*im*)) shows specific expression in the *Bm* PGs (in comparison with its levels in Br-CC-CA complexes (Additional file 11: Table S10 and Additional file 12: Table S11). Even the proposed transcription factor network in *Dm* PGs [4] does not apply for the *Bm* PGs. Identification of gene orthologues proposed to participate in interactions of RG-enriched transcription factors with some of their putative RG targets, based on REDfly and modENCODE data [4, 94, 95], shows that only *Traf4* is expressed in *Bm* PGs while *Snail* expression in RGs [4] is not supported by the RNA-seq data [31] and no orthologue of *Snail* is identified as expressed in *Bm* PGs (Additional file 12: Table S11).

The transcription factor HR4 has been proposed as an example of a factor that shows no transcript enrichment in the *Dm* RG, but appears to be a key regulator of PTTH signalling in both *Bm* and *Dm* [4]. Our results support this notion only within the context of PTTH signalling since *HR4* expression was undetectable until V-6 and had a peak of expression only on V-7, the day after a major secretion of PTTH takes place in *Bm* [36]. Thus, *HR4* expression can serve as a marker of PTTH/Torso transcriptional activation of PGs but its precise role in *Bm* PGs, beyond the PTTH signalling cascade, remains elusive given the complex patterns of expression of the Halloween genes (Fig. 5).

## Conclusions

We believe that the diversity in gene expression we observe in cells of the same tissue from two different insect species is likely to have an underlying biological basis that is intrinsically complex and reflects the evolutionary distance between the two species. Therefore, caution should be exercised when interpreting data on steroid synthesis and secretion from one species as a universal paradigm, when in fact it may be a species-specific paradigm. This rule, though, should not apply to the components of the transduceosome [13, 34] (Table 1) which seems to be the key regulatory mechanism in unravelling the inherent differences between mammalian and insect steroidogenesis.

## Methods

### Animals

The hybrid J106xDAIZO of *Bombyx mori* was used in this study. Hybrid animals were generated from the pure races, J106 and DAIZO, that are bred and maintained in the insect rearing facility of the Department of Biology, Naitonal and Kapodistrian University of Athens. In this hybrid, the 5th instar period lasts about ~ 208 h, the onset of pupal commitment occurs after 60 h (day 3) and the onset of wandering behaviour occurs 144 h (day 6) after the final larval ecdysis. This hybrid has a short period of cocoon spinning that lasts ~ 38 h followed by a period of ~ 26 h before pupal metamorphosis. In this study, each day of the 5th (V) instar is designated with its numerical number (i.e.V-0, V-1 etc.) while the first day of the pupal stage is designated as P-0. Larvae were reared on fresh mulberry leaves under a 12:12-L:D photoperiod at 25 ± 1 °C and 60% relative humidity. Larvae were staged after every larval ecdysis, and the day of each ecdysis was designated as day 0 (V-0). Since larvae mainly moult to the final (5th) instar during the scotophase, all larvae that ecdysed during the scotophase were segregated immediately after the onset of photophase. This time was designated as 0 h of the 5th instar and 4 h later samples of PGs were taken (V-0 samples), while samples of PGs from day 6 (V-6) were taken 144 h later, at the onset of wandering behaviour.

### Quantification of cAMP levels in prothoracic glands

Larvae were anesthetized by submersion in water and PGs were dissected rapidly (~ 2 min/animal) from each larva in sterile saline (0.85% NaCl). The glands were pre-incubated in Grace**’**s medium (Invitrogen) for 15**–**30 min and then each gland was incubated for 30 min in 50 μl of Grace’s medium at 25 ± 1 °C in a well of a 96-well plate (Greiner) with or without either forskolin (50 μM), 3-isobutyl 1-methylxanthine (IBMX) (100 μM), or ionomycin (50 μM) (Calbiochem) for 30 min at 25 ± 1 °C. The cAMP levels in PGs were quantified by an enzyme immunoassay [95] kit according to manufacturer’s instructions (Cayman Chemical Company). Prothoracic glands (*n* = 4–5) were removed after incubation and homogenized in 100 μl acidified ethanol (ethanol/1 N HCl, 100:1 *v*/v). The homogenate was centrifuged (2000 g, 15 min) and the supernatant was removed and saved. The pellet was resuspended in 200 μl ethanol/DW (2:1 v/v) and centrifuged as above. The supernatants were combined, dried in vacuo, and the residue was dissolved in phosphate buffer provided with the kit and acetylation of standards or samples by acetic anhydride was performed according to the instructions in the kit.

### Transcriptomic analysis of *Bombyx mori* PG cells

Prothoracic glands from V-0 and V-6 (onset of wandering stage) of the 5th instar were isolated as described above, meticulously cleared of any associated tissue or debris and total RNA was isolated immediately upon gland removal with TRIzol (Invitrogen) according to the manufacturer’s instructions. The lllumina® mRNA-Seq Sample Prep Kit was used to process the samples according to the manufacturer’s instructions (1,004,898 Rev.D). Briefly, mRNA was isolated from total RNA using oligo-dT magnetic beads. After fragmentation of the mRNA, cDNA synthesis was performed and the resulting cDNA was ligated with the sequencing adapters and amplified by PCR. Quality and yield after sample preparation was measured with the Agilent 2100 Bioanalyzer (Agilent Technologies). The size of the resulting products was consistent with the expected size distribution with a broad peak between 200 and 500 bp on a DNA 1000 chip. A concentration of 17 pM of DNA was used for clustering and DNA sequencing on lllumina cBot and HiSeq2500 (HCS v2.2.58 software) according to manufacturer’s protocols.

### The *Bombyx mori* reference genome

To analyse the raw files from RNA-seq experiments that compared transcript abundance between *Bm* prothoracic glands from V-0 and V-6 of the 5th instar [27, 28] as well as public *Bm* RNA-Seq data from *Bm* PG cells and Br-CC-CA complexes at the wandering stage of the 5th instar, which is equivalent to V-6 of our samples [29, 30] (DDBJ sequence read archive accession number: DRA002282), we constructed a *Bm* reference genome by retrieving sequences assembled in scaffolds and anchored to chromosomes from the public data repository of KAIKObase (http://sgp.dna.affrc.go.jp/pubdata/genomicsequences.html). Contigs of *Bm* genome assembled to scaffolds but not anchored to chromosomes that were less than 20 kb were excluded and the remaining contigs were assembled to a final FASTA file which was used to construct a Bowtie2 [96] (http://bowtie-bio.sourceforge.net/bowtie2/index.shtml) index for subsequent use with TopHat2 [97] and Bowtie2 [96] aligners. Comprehensive gene sets were retrieved again from KAIKObase (http://sgp.dna.affrc.go.jp/ComprehensiveGeneSet/) and a similar procedure as described above was followed to construct a gene file containing genes anchored to chromosomes and additional genes which were inferred in the scaffold sequences so as to construct a final *Bm* gene file in GTF format to supply it to the TopHat2 aligner. From the final GTF file, BED files suitable for visualization in the University of California, Santa Cruz (UCSC) Genome Browser (https://genome.ucsc.edu/) were also constructed.

### Short read mapping

A total of 1.63 Gb from PG samples and 1.45 Gb from Br-CC-CA samples [29, 30] assembled in the FASTQ files containing single-end 36 bp sequence reads, from DDBJ sequence read archive accession number DRA002282, or paired-end 125 bp sequence reads from our own experiments [27, 28], were subjected to quality control using the FastQC package and mapped on the reference genome using TopHat2 [97] with the standard parameters for reads obtained with Illumina platforms and the *--GTF* parameter was supplied with additional transcript annotation data for the *Bm* reference genome as described above. In the case of our paired-end data, we also applied the following: the *--read-gap-length* and *--read-edit-dist* parameters were set to 3 (default is 2) to allow some more freedom in the strictness of the overall alignment procedure since the *Bm* genome is not fully annotated yet. For both datasets, after completing a first round of spliced alignment with TopHat2, reads which failed to map to the reference genome were supplied to the *bedtools bamtofastq* command from the BEDTools suite (https://github.com/arq5x/bedtools2) to create a FASTQ subset of the original raw short reads. These short reads subset was subjected to a second round of unspliced alignment with Bowtie2 in sensitive mode (options applied: *--local --very-sensitive-local --maxins 1000 --dovetail*) to allow mapping of part of this subset back to the reference genome. Mapping of this subset back to the reference genome occurred when a sufficient and continuous proportion of each read was successfully aligned. This procedure allowed for the alignment of paired-end reads located quite further than the average pair distance. These two rounds of alignment procedure led to increased alignment rates.

### Cufflinks analysis of *Bombyx mori* PG cells transcripts

The raw reads of our RNA-seq data have been deposited into the NCBI Short Read Archive (SRA, http://www.ncbi.nlm.nih.gov/sra/) under accession number SRP062258 [27]. This record combines 3 biological replicates from V-0 and 3 biological replicates from V-6 with accession numbers SAMN03978782 and SAMN03978783, respectively. To compare and complement the results of the differential expression analysis on our RNA-seq data performed using PANDORA [27, 28] as well as explore potential differentially expressed isoforms, we used the Cufflinks pipeline [98]. Specifically, we first reassembled and quantified the *Bm* transcriptome for each sample using the Cufflinks command with parameters *--multi-read-correct*, *−-max-mle-iteration 10,000*, *−-max-bundle-frags 100,000*, *−-overhang-tolerance 10*, *−-max-multiread-fraction 0.5*, *−-no-faux-reads --upper-quartile-norm*. In addition, we provided a guide for the transcriptome assembly of Cufflinks using a GFF file we assembled from the procedure described in the previous section through the cufflinks option *--GTF-guide*. Subsequently, we merged the assembled and quantified transcriptomes using the *cuffmerge* command. Finally, we executed *cuffdiff* for the contrast V-6 versus V-0 with the following arguments: *--multi-read-correct*, *−-max-mle-iteration 10,000*, *−-max-bundle-frags 100,000*.

### Statistical analysis

In order to perform differential expression analysis of the RNA-Seq data, we used the Bioconductor [99] package metaseqR with the PANDORA [39] method which has been shown to improve the overall accuracy by optimising the trade-off between true positives and false hits in differential expression analysis through the systematic combination of multiple analysis algorithms. Specifically, and regarding the metaseqR workflow, the BAM files, one for each RNA-seq sample [27, 29, 30], were summarised to a gene read counts table, using the Bioconductor package GenomicRanges [100] and the *Bm* genes derived as described above. Prior to the statistical testing procedure, the gene read counts were filtered for possible artefacts that could affect the subsequent statistical testing procedures. Genes presenting any of the following were excluded from further analysis: i) genes whose average reads per 100 bp was less than the 25th quantile of the total normalised distribution of average reads per 100 bp, ii) genes with read counts below the median read counts of the total normalised count distribution. The resulting gene counts table was subjected to differential expression analysis for the contrasts Br-CC-CA versus PG cells [29], using the Bioconductor packages DESeq [101], edgeR [102], limma [103], NBPSeq [104], NOISeq [105] and baySeq [106]. To combine the statistical significance from multiple algorithms so as to optimise the trade-off between true positives and false hits, we applied the PANDORA [39] weighted *p*-value method across all results. In the final read counts table, which was used for differential expression analysis, each row represented one gene, each column one RNA-Seq sample and each cell the corresponding read counts associated with each row and column.

### RNA-seq data visualisation

To create a UCSC Genome Browser visualization track data hub, BAM files resulting from the alignment procedure, of our RNA-seq data [27] and that of another research group [29, 30], were converted to BED format (https://genome.ucsc.edu/FAQ/FAQformat.html#format1) using the *bedtools bamtobed* command from the BEDTools suite (http://bedtools.readthedocs.org/en/latest/index.html#) with the *-split* option to report RNA-seq reads split by the TopHat2 algorithm as separate alignments, referred hereafter as “tags”. The RNA signal from these files was extracted by reformatting them in BedGraph (http://genome.ucsc.edu/goldenPath/help/bedgraph.html) format using the *bedtools genomecov* command from the BEDTools suite with the *-bg* option and then to bigwig (https://genome.ucsc.edu/goldenpath/help/bigWig.html) format using the *bedGraphToBigWig* program supplied by UCSC. The bigWig tracks were visualised in a custom UCSC Genome Browser track data hub, hosting the *Bm* (bmori2) reference genome and the normalised (total signal of 10^10^) RNA-seq samples. Two separate track data hubs were generated. One for our RNA-seq data [27, 28] for V-0 and V-6 of the 5th instar at (http://epigenomics.fleming.gr/tracks/hs_trackhubs/ekpa_dedos_2/hub.txt) and another for the RNA-seq data generated by another research team [29, 30] (DDBJ Sequence Read Archive Accession number: DRA002282) which compares transcript abundance between *Bm* PGs and Br-CC-CA complexes at the onset of wandering stage at (http://epigenomics.fleming.gr/tracks/hs_trackhubs/ekpa_dedos_1/hub.txt). Each link must be pasted in the My Hubs tab of the UCSC Genome Browser application.

### Data assembly and bioinformatic analysis

Τo identify candidate coding regions within transcript sequences as predicted by Cufflinks analysis, we used the TransDecoder algorithm [37] (https://github.com/TransDecoder/TransDecoder/wiki). Specifically, we used TransDecoder utilities to convert the assembled cufflinks transcriptome file to a FASTA file containing the corresponding cDNAs and input this file to TransDecoder with standard parameters. Subsequently, we applied standard Blast of the TransDecoder identified longest ORFs output against the Uniprot database (with parameters *-max_target_seqs 1 -evalue 1e-5*) and against the Trembl database (with parameters *-max_target_seqs 1 -evalue 1e-6*). We also looked for domain homologies against the Pfam database using again TransDecoder facilities. The final protein coding potential was assessed using the prediction algorithm of TransDecoder and the results of the previous steps. To produce a comprehensive protein dataset that was used to assign orthologues, the TransDecoder output was subjected to Blastp analysis on NCBI against the *Bm* annotated genes (annotation 102) https://www.ncbi.nlm.nih.gov/genome/annotation_euk/Bombyx_mori/102/. Results were filtered to retain the best hit and % identity for each protein sequence. Then, the Cufflinks, TransDecoder [37] (https://github.com/TransDecoder/TransDecoder/wiki), PANDORA [27, 28] and Blastp analysis files were integrated into a single file that was used to further analyse our results. In addition, we re-analysed, as described above, and integrated in our final analysis the raw files from RNA-seq data that compared transcript abundance between *Bm* PG cells and Br-CC-CA complexes at the wandering stage of the 5th instar [29, 30]. Once tabulated this dataset was used to define as PG-specific genes that had: 1) meta *p*-value < 0.05, and 2) meta FDR value < 0.05, and 3) expression ratio PG/Br > 1, and 4) Br RPGM <median value (0.4902) and 5) PG RPGM value > 0.0259 (i.e. the value found to be the lowest for PG expression by qPCR (see below), and 6) Cufflink values above those found for expressed genes (see below). The final list contained 272 PG-specific genes (see Additional file 11: Table S10).

We also used RNA-seq data available from the wandering stage of *Drosophila melanogaster* [31] and annotated data of transcripts specifically expressed in the *Dm* RGs during the final larval instar [4].

With these four datasets, we could define gene expression patterns in the wandering stage of both insect species and also identify the genes that are predominantly, although not exclusively, expressed in PG cells. To determine the tissue specificity of genes that are up-regulated and down-regulated by prothoracicotropic hormone stimulation of PG cells, we analysed and annotated RNA-seq data previously described [4]. All these datasets were integrated with phenotypic data from *Dm* RG-specific RNAi experiments [22, 32] coupled with assignment of gene orthologues between the two species using OrthoDB [107], g:Orth [40] and OrthoMCL [108].

KEGG pathway maps (http://www.genome.ad.jp/kegg/pathway.html) where available for *Bm* [109, 110] or *Dm* [110] were used to identify proteins that are components of signalling pathways and examine their presence in the various datasets. For AC encoding genes present in *Bm* genome, *Dm* AC protein sequences [111] were searched by Blastp against the *Bm* RefSeq annotated proteins (annotation 102). Hits were downloaded, manually curated to identify partial sequences, alternatively spliced variants and duplicate entries and then validated against the *Bm* genome database (http://sgp.dna.affrc.go.jp/KAIKObase/) and annotated to their corresponding gene(s). Closely related *Bm* proteins were also retrieved using a combination of literature searches and available databases such as the UniprotKB (http://www.uniprot.org), the SilkDB database (http://metazoa.ensembl.org/Bombyx_mori/Info/Index) and tBLASTN queries against the KAIKOBLAST server (http://sgp.dna.affrc.go.jp/KAIKObase/) to identify and exclude any possibility that a protein of interest was left undetected. Selected hits were then searched using the profile hidden Markov model on HMMER (http://www.ebi.ac.uk/Tools/hmmer/) to identify the Pfam-A domains present on each protein sequence. Additional domain analysis of the retrieved protein sequences was carried out in PROSITE (http://au.expasy.org/prosite/) and InterPro (http://www.ebi.ac.uk/interpro/). Signal peptide and transmembrane domains were analysed by SignalP4.1 (http://www.cbs.dtu.dk/services/SignalP/) and TMHMM server v.2.0 (http://www.cbs.dtu.dk/services/TMHMM/), respectively. Exactly the same process, as the one described above, was followed for 3′, 5′ cyclic nucleotide phosphodiesterase encoding genes (hereafter referred to as phosphodiesterases(PDEs)) present in *Bm* genome using *Dm* PDE protein sequences [112], for protein kinase C (PKC) encoding genes present in *Bm* genome using *Dm* PKC protein sequences [113], and for phospholipase C encoding genes present in *Bm* genome using *Dm* PLC protein sequences including the phospholipase C X-domain containing protein sequences [114]. For cytochrome P450 (CYP) analysis, we used sequences available from the cytochrome P450 homepage [115] (http://drnelson.uthsc.edu/silkworm.pub.htm) as well as results presented in previous studies [70, 71]. By combining the available sequences from these sources and validating them against the GenBank database, we conclude that there are 82 cytochrome P450 genes in the *Bm* genome. Our results largely agree with previous studies [70, 71] apart from the following cases: CYP6AE3P [70] protein sequence is identical to CYP6AE21 [115] and CYP6AE2 [70] protein sequence is identical to CYP6AE8 [115]. The full length sequences of proteins encoded by *CYP333un1*, *CYP340A5P* and *CYP340un1*, that were previously reported to be pseudogenes [70], were recovered (see Additional file 20: Table S17) and these genes are not predicted to be pseudogenes. *CYP340A1*-*de9b* [70] is part of a misplaced genomic scaffold and *CYP4G45* [70] is probably an erroneous entry as suggested in (http://drnelson.uthsc.edu/silkworm.pub.htm) [115]. The 82 annotated protein sequences were searched using HMMER3 (http://hmmer.janelia.org/) with the default parameters. As described before [71], the insect CYP genes code for proteins of approximately 500 amino acids each having five conserved motifs: the helix C motif (WxxxR), the helix I (oxygen binding) motif (Gx[E/D]T[T/S]), the helix K motif (ExLR), the PERF motif (PxxFxP[E/D]RF) and the heme-binding motif (PFxxGxRxCx[G/A]) all found in the sequences we annotated.

To annotate the secondary structure of selected domains shown in figures, the putative three-dimensional structure of the protein indicated in figure legends was predicted at the automated comparative protein modelling server (Swiss-Model) (https://www.swissmodel.expasy.org/) with the optimized mode using the coordinates of the best model for each protein group available from the Brookhaven Protein Database (BPD) [116].

For Gene Ontology (GO) term enrichment analysis, the Gene Ontology Consortium term description and hierarchical ranking (http://amigo2.berkeleybop.org/amigo/landing) was adopted. We used g:Cocoa [40] to identify clusters of biological processes and molecular functions that were highly represented (*p*-value) in our datasets with *Bombyx mori* as the species and default settings.

### Sequence alignments and phylogenetic analysis

Protein sequences were assembled from GenBank or Flybase (see above) and aligned using the software MSAProbs [117] v0.9.7 with default parameters. In each of the five protein datasets we examined, phylogenetic relationships were inferred from maximum likelihood and Bayesian inference. We used Prottest v3.4.2 [118] to identify the appropriate phylogenetic model for each of the 5 different alignments. According to Bayesian Information Criterion (BIC) framework, the best fit model for ACs, was LG + I + G + F, which uses a general amino acid replacement matrix [119] with a proportion of invariable sites (+I) [120], a gamma distribution for modelling the rate heterogeneity (+G) [121], and empirical amino acid frequencies (+F) [122]. Likewise, the best fit model for PDEs was VT + I + G + F which uses a general amino acid replacement matrix [123], the best fit model for PKCs was VT + I + G, the best fit model for PLCs was LG + I + G and the best fit model for CYPs was LG + I + G + F. MrBayes 3.2.6 [124] was used for Bayesian inference tree calculation on 2 parallel sets of 4 chains (3 hot and 1 cold) run for 100,000 generations until convergence was reached (‘average standard deviation of split frequencies’ < 0.01). Temperature heating parameter was set to 0.2 (temp = 0.2) and burn-in was set to 25% (burninfrac = 0.25). The average standard deviation of split frequencies was 0.000000 for the analysis of ACs, PDEs, PKCs and PLCs and 0.007888 for CYPs. Maximum likelihood trees were produced with RAxML 8.2.3 using the LG4X PROTGAMMA model [125] and 1000 bootstrap replicates [126]. All analyses were run on the CIPRES server at the San Diego Supercomputer Center (SDSC). Trees were visualized and annotated in figtree v1.4.3 (http://tree.bio.ed.ac.uk/software/figtree/).

### Total RNA isolation and cDNA synthesis from prothoracic glands

Prothoracic gland isolation was carried out as described above, glands were meticulously cleared of any associated tissue or debris and total RNA was isolated immediately with TRIzol (Invitrogen) according to manufacturer’s instructions (*n* = 7 for each day of the investigated developmental period). Integrity of total RNA from each sample was determined using gel electrophoresis and RNA quality was determined by measuring the absorbance at 260 and 280 nm (A260/280 of all samples > 1.9). First strand cDNA was synthesized from 2 μg total RNA with 200 U Superscript®III reverse transcriptase (Invitrogen) in 20 μl reaction volumes using an oligo_20_ primer (Invitrogen) according to manufacturer’s instructions. The resulting cDNA was diluted with nuclease-free water (Thermo Fisher Scientific) before use in quantitative PCR.

### Quantitative PCR analysis of *Bombyx mori* genes

Quantitative real-time PCR was carried out with the SYBR® Green dye in 96-well PCR micro plates (Applied Biosystems) on a 7500 Real-Time PCR System (Applied Biosystems). Fluorescence emission of the products and subsequent calculations were carried out with the Sequence Detection System software v2.0.6 (Applied Biosystems). The reaction mixture (10 μl total volume per well) included 20 ng cDNA, 0.8 μl nuclease-free water (Thermo Fisher Scientific), 5 μl Kapa SYBR® Fast Universal 2X qPCR Master Mix (Kapa Biosystems), 0.2 μl of 50× Rox Low passive reference dye (Kapa Biosystems) and primers at a final concentration of 200 nmol/l. Reactions (*n* = 3–7) to amplify 230–270 bp amplicons were performed under the following conditions: 95 °C for 3 min as an initial step followed by 40 cycles of 95 °C for 15 s and 60 °C for 60 s. After amplification, dissociation curves were produced (60 °C to 95 °C at a heating rate of 0.1 °C/sec and acquiring fluorescence data every 0.3 °C) to discriminate the main reaction products from other nonspecific ones or primer-dimers and PCR products were subjected to electrophoresis on 2% *w*/*v* agarose gels to corroborate the presence of a unique amplicon (see Additional file 25: Table S19 for detailed description). Each qPCR run always included a no-cDNA template control and reverse transcription negative controls. For each of the replicates (*n* = 7) genomic DNA as a qPCR template was also analysed. All the aforementioned negative controls gave no detectable quantification cycle (*C*_*q*_) value, proving the lack of any contamination or nonspecific signal. Absolute quantification analysis via a standard curve approach was utilized to calculate the transcript levels of 650 genes (see legend of Additional file 7: Table S7). Serial dilutions (4 to 4 × 10^6^ copies/reaction) of known concentration of *Bombyx mori Torso* (*BmTorso*) in plasmid *pBRAcPA* [41] (a generous gift from Dr. Michael O’Connor, University of Minnesota, USA), linearised with *NheI*, was used in each qPCR run to construct the standard curve(s). Concentration of the standard solutions of the plasmid was determined by spectrophotometry and was converted to copy numbers per microliter using the following formula: Copy number/μl = 6.023 × 10^23^ copies/mol × DNA concentration (g/μl)/molecular weight (g/mol). Standard curves were constructed by plotting the threshold cycle (C*q*; ΔRn = 0.25) values against the initial copy number of *BmTorso* containing plasmid. Copy numbers of transcripts in the samples were calculated by interpolating the C*q* value of the sample within the generated standard curve(s). We conducted validation experiments to test the requirements for applying the above methodology. We estimated reaction efficiency (*E*) with the formula, *E*% = [− 1 + 10^(− 1/slope)^] × 100 using dilution series of sample cDNA, incorporating several orders of magnitude (100–0.1 ng) and *C*_*q*_ values were plotted against log_10_[cDNA quantity]. Primers used for qPCR are listed in Additional file 26: Table S20 and the MIQE guidelines-adopted [127] complete protocols of the qPCR assays are described in Additional file 25: Table S19 [27, 28]. All primers were designed with an online tool (http://primer3plus.com/cgi-bin/dev/primer3plus.cgi) using the following custom settings: 1) amplicons size range 230–270 bp, 2) primer size minimum 18 bases, optimum 20 bases, maximum 23 bases, 3) primer *Tm* minimum 59 °C, optimum 60 °C, maximum 62 °C, maximum *Tm* difference 1 °C, 4) Primer GC% minimum 30, optimal 50, maximum 80, and the other settings were left to default values. Among the various combinations of primers returned by the online tool, the pair that combined the following criteria was selected: 1) minimal penalty value, 2) amplicon at the 3′ end of the cDNA sequence, 3) unique hit in NCBI Primer-Blast (http://www.ncbi.nlm.nih.gov/tools/primer-blast/index.cgi?LINK_LOC=BlastHome) webtool using the RefseqmRNA database and *Bombyx mori* as the species, 4) unique hit in the KAIKObase website using Blastn.

## Additional files

Additional file 1: **Table S1.** Tabulation of 61,260 tests carried out by Cufflinks on the raw transcriptomic data. In cases where a gene model was included between the examined locus, this is indicated using the nomenclature of KAIKObase (http://sgp.dna.affrc.go.jp/KAIKObase/) or it is left empty. Using this criterion, we identified 10,775 unmapped loci. (XLSX 5956 kb)

Additional file 2: **Table S2.** Tabulation of 43,242 tests carried out by TransDecoder on the Cufflinks output. TransDecoder settings were adjusted to filter out ORFs encoding < 100 amino acid peptides. Columns E to I are the TransDecoder output, columns J to Q are the Cufflinks output and columns R to AC are the results of the Blastp analysis against the *Bm* protein reference sequences (NCBI annotation release 102). In cases where a gene model was included between the test ID coordinates (Column C), this is indicated using the nomenclature of KAIKObase (http://sgp.dna.affrc.go.jp/KAIKObase/) or it is left empty. Using this criterion, we identified 3579 unmapped loci. (XLSX 13932 kb)

Additional file 3: **Table S3.** Tabulation of 16,550 unique loci based on the TransDecoder and Cufflinks output. Data from Additional file 2: Table S2 was manually annotated to filter out duplicate entries based on locus and protein sequence. Columns E to H are the TransDecoder output, columns I to P are the Cufflinks output, columns R to Y are the Bowtie2 output [28], columns Z and AA are the results of the Blastp analysis against the *Bm* protein reference sequences (NCBI annotation release 102) and columns AB to AE are the results of the g:Orth [40] and OrthoDB [107] output. In cases where a gene model was included between the test ID coordinates (Column D), this is indicated using the nomenclature of KAIKObase (http://sgp.dna.affrc.go.jp/KAIKObase/) or it is left empty. Using this criterion, we identified 2265 unmapped loci. (XLSX 8960 kb)

Additional file 4: **Table S4.** Tabulation of 51,035 tests carried out by Cufflinks to identify splicing events that lead to differential gene expression in *Bombyx mori* PGs between V-0 and V-6 of the 5th instar. In cases where a gene model was included between the test ID coordinates (Column C), this is indicated using the nomenclature of KAIKObase (http://sgp.dna.affrc.go.jp/KAIKObase/) or it is left empty. Using this criterion, we identified 12 unmapped loci among the 231 differentially spliced loci. (XLSX 2546 kb)

Additional file 5: **Table S5.** Tabulation of the 231 differentially spliced loci identified by Cufflinks in *Bombyx mori* PGs between V-0 and V-6 of the 5th instar. In cases where a gene model was included between the test ID coordinates (Column C), this is indicated using the nomenclature of KAIKObase (http://sgp.dna.affrc.go.jp/KAIKObase/) or it is left empty. Each test was annotated with its corresponding data from the Bowtie2 output [28] (columns U to AB) and the results of the Blastp analysis against the *Bm* protein reference sequences (NCBI annotation release 102) (columns AC and AD). (XLSX 132 kb)

Additional file 6: **Table S6.** Tabulation of 98 unique differentially spliced loci based on the TransDecoder and Cufflinks output. Data from Additional file 5: Table S5 was manually annotated to filter out duplicate entries based on locus and protein sequence. In cases where a gene model was included between the test ID coordinates (Column C), this is indicated using the nomenclature of KAIKObase (http://sgp.dna.affrc.go.jp/KAIKObase/) or it is left empty. Each test was annotated with its corresponding data from the Bowtie2 output [28] (columns R to Y) and the results of the Blastp analysis against the *Bombyx mori* protein reference sequences (NCBI annotation release 102) (columns Z and AA). (XLSX 131 kb)

Additional file 7: **Table S7.** Tabulation of 650 loci of *Bombyx mori* genone whose transcript abundance in PG cells was used to determine the genes expressed in these cells. Data from Bowtie2 (column F and G) [27, 28] and Cufflinks (column D and E) was integrated with qPCR results and used to plot Fig. 1d and e. (XLSX 94 kb)

Additional file 8: **Figure S1. A:** Venn diagram comparing the identified genes (see Additional file 3: Table S3), the differentially expressed genes (blue) (see Additional file 3: Table S3) between V-0 and V-6 and the alternatively spliced genes (orange) (see Additional file 6: Table S6) between V-0 and V-6. **B:** Venn diagram comparing the expressed genes on V-0 and V-6 (see Additional file 3: Table S3), the differentially expressed genes (blue) (see Additional file 3: Table S3) between V-0 and V-6 and the alternatively spliced genes (orange) (see Additional file 6: Table S6) between V-0 and V-6. (TIFF 64679 kb)

Additional file 9: **Table S8.** Tabulation of 10,744 orthologous genes between *Bombyx mori* and *Drosophila melanogaster*. Data was integrated from OrthoDB [107], g:Orth [40], OrthoMCL [108] and manually curated, literature-based data. The final dataset was enriched with the phenotypic data derived from gene silencing by RNAi in an RG-specific manner [32] (column P to S) and RNA-seq data from *Dm* [31] (column T to V). (XLSX 2049 kb)

Additional file 10: **Table S9.** Analysis of RNA-seq data from PGs and Br-CC-CA at the wandering stage of *Bombyx mori* using PANDORA [39]. RNA-seq data was downloaded from DDBJ Sequence Read Archive (Accession number: DRA002282) [29, 30] and analysed to identify PG-specific genes (see Methods for details). The raw output of PANDORA is tabulated to include 15,726 *Bm* gene models using the nomenclature of KAIKObase (http://sgp.dna.affrc.go.jp/KAIKObase/). (XLSX 3792 kb)

Additional file 11: **Table S10.** Tabulation of 272 PG-specifc gene models using the nomenclature of KAIKObase (http://sgp.dna.affrc.go.jp/KAIKObase/). Data included in this table is shortlisted from Additional file 10: Table S9 and used in Fig. 2b and c (see text for details). (XLSX 337 kb)

Additional file 12: **Table S11.** Tabulation of 1617 data points used to construct Fig. 2b. Data was derived from [4, 27–32] (see text for details). (XLSX 647 kb)

Additional file 13: **Table S12.** Tabulation of 76 gene models that were down-regulated by PTTH and 173 gene models that were up-regulated by PTTH based on data from [4]. Data derived from [4] was annotated using the nomenclature of KAIKObase (http://sgp.dna.affrc.go.jp/KAIKObase/) and enriched with our Bowtie2 data [27], the Cufflinks data (this study) and *Dm* orthologue data as presented in [4]. (XLSX 167 kb)

Additional file 14: **Table S13.** Integration of *Bombyx mori* orthologues with the *Drosophila melanogaster* RG-specific genes affected by Ras^V12^ or Torso-RNAi phenotypes as described [4]. The data shown in the table corresponds to data presented on Fig. 5i and j of [4]. (XLSX 90 kb)

Additional file 15: **Table S14.** Tabulation of 350 loci with > 1,000,000 short reads per locus based on the Cufflinks output. Data from Additional file 3: Table S3 with the indication HIDATA (column I) is shown. In cases where a gene model was included between the test ID coordinates (Column D), this is indicated using the nomenclature of KAIKObase (http://sgp.dna.affrc.go.jp/KAIKObase/) or it is left empty. Each test was annotated with its corresponding data from the Bowtie2 output [28] (columns R to Y) and the results of the Blastp analysis against the *Bm* protein reference sequences (NCBI annotation release 102) (columns Z and AA). (XLSX 263 kb)

Additional file 16: **Table S15.** Tabulation of 1894 gene models, using the nomenclature of KAIKObase (http://sgp.dna.affrc.go.jp/KAIKObase/), whose *Drosophila melanogaster* orthologues had an obvious phenotype (column AT) when silenced by RG-specific RNAi. Data from [32] was used to identify the *Bm* orthologues of *Dm* genes using OrthoDB [107]. The *Bm* orthologues were then annotated with our LC-MS/MS data [28] (column F to R), our Bowtie2 data [27] (column S to Z), our Cufflinks data (column AA to AN), and the phenotypic data [32] (column AQ to AT). Data from this table is presented in Fig. 2f. (XLSX 1346 kb)

Additional file 17: **Table S16.** Adenylate cyclases, phosphodiesterases, protein kinases C and phospholipases C of *Bombyx mori* and *Drosophila melanogaster*. Annotation for each gene was enriched with data from [4, 27–32]. The sequence of each protein used to construct the posterior probability trees is shown in column AN and AO for *Bm* and *Dm*, respectively. (XLSX 130 kb)

Additional file 18: **Figure S2.** *Bombyx mori* adenylate cyclases and 3′, 5′ cyclic nucleotide phosphodiesterases. **A:** Secondary structure and multiple sequence alignment of the C1 and C2 domains of *Bm* ACs based on sequence similarities with mammalian ACs [48, 49]. Secondary structure prediction of BmAC1L was carried out using Swiss model protein structure homology-modelling server [116] and is shown on top of each alignment. Arrows indicate β-sheets and cylinders indicate α-helixes. Forskolin interacting residues are highlighted in green based on data from mammalian ACs [48, 49]. ATP binding residues are highlighted in blue based on data from mammalian adenylate cyclases [48, 49]. Boxed residues indicate the change of Serine to Alanine in BmAC8L and BmAC5L that probably confers insensitivity to forskolin [50]. The name of each adenylate cyclase is based on sequence similarity of each protein to the human adenylate cyclases (i.e. BmAC1L: *Bombyx mori* Adenylate Cyclase 1 (human adenylate cyclase 1)-Like). **B:** Graphical representation of the Pfam domains of *Bm* ACs. Pfam domains were visualized via HMMER https://www.ebi.ac.uk/Tools/hmmer/search/phmmer [128]. The length of each protein and the location of transmembrane domains is indicated. **C:** Secondary structure and multiple sequence alignment of the catalytic domain of *Bm* PDEs based on sequence similarities with mammalian PDEs [52–54]. Secondary structure prediction of BmPDE1L was carried out using Swiss model protein structure homology-modelling server [116] and is shown on top of each alignment. Cylinders indicate α-helixes. IBMX interacting residues are highlighted in blue [55]. Residues contacting the AMP ligand or otherwise involved in the catalytic mechanism are highlighted in orange based on sequence similarities with mammalian phosphodiesterases [52–54]. The name of each phosphodiesterase is based on sequence similarity of each protein to the human phosphodiesterases (i.e. BmPDE4L: *Bombyx mori* PhospoDiEsterase 4 (human phosphodiesterase 4)-Like). **D:** Graphical representation of the Pfam domains of *Bm* PDEs. Pfam domains were visualized via HMMER https://www.ebi.ac.uk/Tools/hmmer/search/phmmer [128]. The length of each protein is indicated. (TIFF 450352 kb)

Additional file 19: **Figure S3.** *Bombyx mori* PKCs, PLCs and PI-PLC X dcps. **A:** Upper panel: Secondary structure and multiple sequence alignment of the C1 domain of three PKCs and the *Bos taurus* PKCα (BtcPKCα) C1 domain. Residues highlighted in green bind phorbol esters or are implicated in the structural integrity of the domain as described [62]. Boxed Arginine residues have been shown to abolish phorbol ester binding to PKC [63]. Single letters below the alignment indicate the conserved motif of the C1 domain HX_12_CX_2_CX_n_CX_2_CX_4_HX_2_CX_7_C [64]. Lower panel: Secondary structure and multiple sequence alignment of the catalytic domain of the four identified *Bm* PKCs. Residues highlighted in orange are the ATP binding motif [65] and residues highlighted in blue are critical phosphorylation sites based on sequence similarities with mammalian PKCs as described [65]. The name of each PKC is based on sequence similarity of each protein to the human PKCs (i.e. BmcPKCα: *Bombyx mori* Conventional PKCα (human PKCα). **B:** Graphical representation of the Pfam domains of *Bm* PKCs, PKN and PKD. Pfam domains were visualized via HMMER https://www.ebi.ac.uk/Tools/hmmer/search/phmmer [128]. **C:** Secondary structure and multiple sequence alignment of the region encompassing the catalytic site of the four identified *Bombyx mori* PLCs based on sequence similarities with the rat PLCδ1 [67, 68]. Residues essential for the catalytic activity of PLCs are highlighted in blue based on sequence similarities with the rat PLCδ1 [67, 68]. The region of BmPLCβ1 and BmPLCβ4 that aligns with the human PLCβ3 region that was shown to interact with Gαq [69] are highlighted in green. The name of each PLC and PI-PLC X dcp is based on sequence similarity of each protein to the human PLC and PI-PLC X dcps (i.e. BmPLCβ1: *Bombyx mori* PhosphoLipase Cβ1 (human PLCβ1). **D:** Graphical representation of the Pfam domains of *Bombyx mori* PLCs and PI-PLC X dcps. Pfam domains were visualised via HMMER https://www.ebi.ac.uk/Tools/hmmer/search/phmmer [128]. (TIFF 450898 kb)

Additional file 20: **Table S17.** Cytochrome P450 genes of *Bombyx mori*. Annotation for each gene was enriched with data from [27, 28]. The sequence of each protein used to construct the posterior probability tree (Additional file 21: Figure S4.) is shown in column AA. (XLSX 136 kb)

Additional file 21: **Figure S4.** Posterior probability tree of *Bombyx mori* cytochrome P450 (CYP) proteins. The short-chain dehydrogenase/reductase protein Shroud [44] was used as an outgroup. Single asterisks indicate genes identified as expressed on V-0 or V-6 of the 5th instar of *Bm* by Cufflinks. Double asterisks indicate genes whose expression in *Bm* PGs has been established by other research groups. Nomenclature of CYP genes is based on [70]. Numbers indicate the posterior probability score (%). (TIFF 394527 kb)

Additional file 22: **Figure S5.** Circos plot comparing the expression profiles of Halloween genes and other ecdysteroidogenesis-related genes during the final larval instar and the first day of the pupal stage of *Bm*. Percentage values are related to C_*Τ*_ values determined by qPCR. See Additional file 7: Table S7 for details. (TIFF 182103 kb)

Additional file 23: **Figure S6.** Expression profile of *BmHR4* during the final larval instar and the first day of the pupal stage of *Bombyx mori*. (TIFF 24374 kb)

Additional file 24: **Table S18.** Integration of *Bombyx mori* plasma membrane receptor expression data in PG cells with RNA-seq data from *Drosophila melanogaster* orthologous genes. Our previous analysis results of plasma membrane receptors expressed in PG cells [27] was integrated with our Cufflinks data and with expression data from *Dm* [31]. Orthology annotation was based on one(*Bombyx mori*) to all (*Drosophila melanogaster*) using OrthoDB [107]. (XLSX 87 kb)

Additional file 25: **Table S19.** Checklist of compliance with the MIQE guidelines [127]. (XLSX 17 kb)

Additional file 26: **Table S20.** List of primers used to determine transcript abundance of the 650 genes shown in Additional file 7: Table S7. (XLSX 94 kb)

## Abbreviations

20E: 20-hydroxyecdysone
3DE-3α-Red: 3-DehydroEcdysone-3α-Reductase
*5-HT1A*: *5-hydroxytryptamine (serotonin) receptor 1A*
AC: Adenylate cyclase
ACAT: Acyl-CoA cholesterol acyltransferase
ACBD1(DBI): Diazepam binding inhibitor
ACBD3: Acyl-CoA binding domain containing 3
ACOT2/3: Acyl-CoA thioesterase 2
ACSL4: Acyl-CoA synthetase 4
AKR2E4: Aldo Keto Reductase 2E4
ANT: Adenine nucleotide transporter
ATAD3: ATPase family AAA domain-containing protein 3
*babo*: *Baboon*
*Bm*: *Bombyx mori*
BPD: Brookhaven Protein Database
*Cam*: *Calmodulin*
cAMP: Cyclic AMP
CYP: Cytochrome P450
dcp: Domain-containing protein
DDBJ: DNA Data Bank of Japan
*Dib*: *Disembodied*
*Dm*: *Drosophila melanogaster*
*E75*: *Ecdysone-induced protein 75*
*EcR*: *Ecdysone Receptor*
EO: Ecdysone Oxidase
ER: endoplasmic reticulum
FDX: ferredoxin
FDXR: ferredoxin reductase
FPKM: Fragments Per Kilobase of transcript per Million of mapped reads
*ftz-f1*: *fushi tarazu transcription factor 1*
*gce*: *Germ cell-expressed bHLH-PAS*
*HR*4: *hormone receptor 4*
*HR*46: *hormone receptor-like 46*
IBMX: 3-isobutyl 1-methylxanthine
IDH2: Isocitrate dehdydrogenase 2
IMM: inner mitochondrial membrane
*InR*: *Insulin Receptor*
LONP1: Mitochondrial lon protease homolog
MAM: mitochondria-associated membrane
MDH2: malate dehydrogenase 2
*mdy*: *Midway*
*Met*: *Methoprene-tolerant*
MLN64: Metastatic lymph node 64
*nobo*: *Noppera-bo*
*Npc-1a*: *Niemann Pick C type 1a*
*Npc-1b*: *Niemann Pick C type 1b*
*Nvd*: *Neverland*
OMM: outer mitochondrial membrane
OPA1: optic atrophy type 1
PDE: Phosphodiesterase
PG: Prothoracic Gland
*Phm*: *Phantom*
*PI3K*: *Phosphotidylinositol 3 kinase*
PKA: protein kinase A (cAMP-dependent)
PKC: Protein kinase C
PLC: Phospholipase C
*put*: *punt*
RG: Ring Gland
RPGM: Reads Per Gene Model
*RpS6*: *Ribosomal protein S6*
*Sad*: *Shadow*
*Shd*: *Shade*
*Smad2*: *Smad on X*
*Spo*: *Spook/Spookier*
*Sro*: *Shroud*
StAR: Steroidogenic Acute Regulatory protein
*Start1*: Orthologue of Steroidogenic Acute Regulatory protein encoding gene
*Tor*: *Target of Rapamycin*
*TSC1*: *tuberous sclerosis complex gene 1*
*TSC2*: *tuberous sclerosis complex gene 2*
TSPO: translocator protein (18-kDa)
*usp*: *ultraspiracle*
VDAC: voltage dependent anion channel
