# Supplementary figures and images for: Refining a steroidogenic model: an analysis of RNA-seq datasets from insect prothoracic glands
Source: BMC Genomics. 2018 Jul 13;19:537. doi: 10.1186/s12864-018-4896-2 (PMC6045881; doi:10.1186/s12864-018-4896-2)

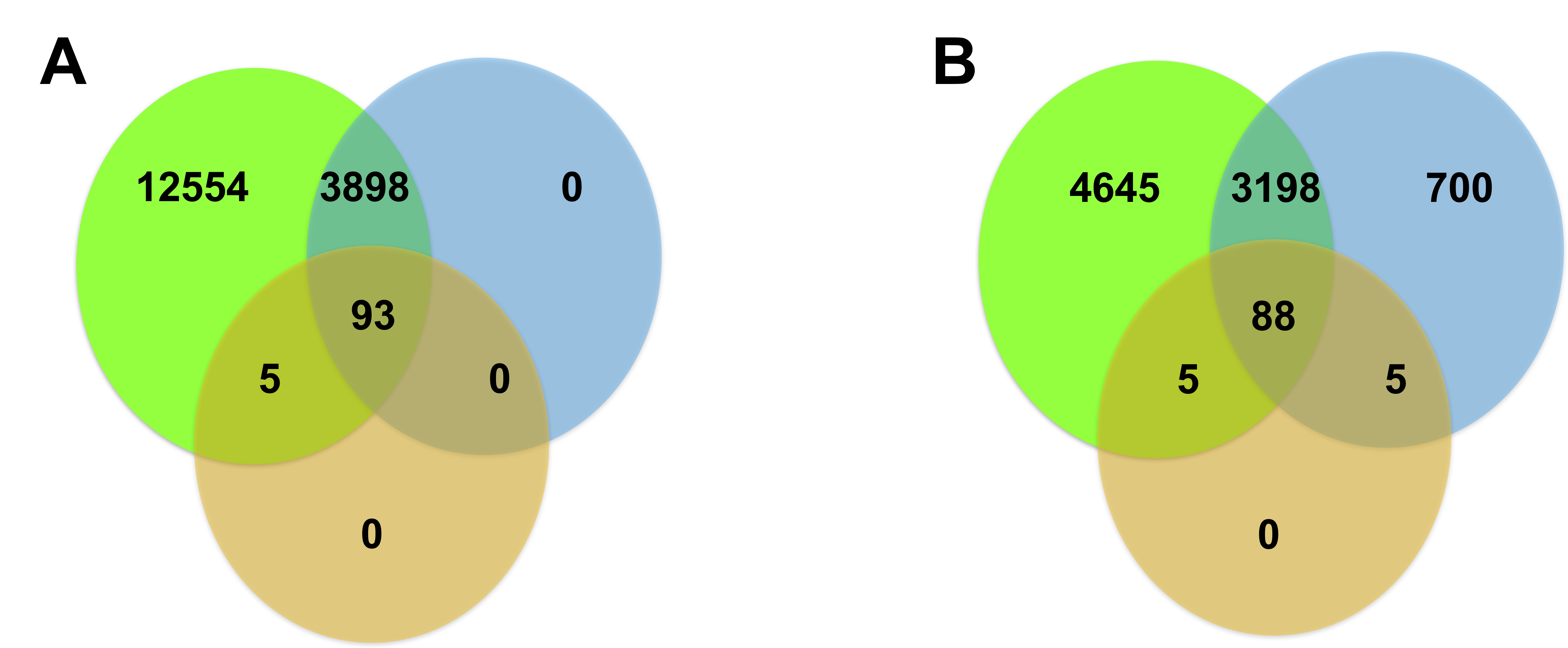

Supplement: Supplementary file 8 — Figure S1. A: Venn diagram comparing the identified genes (see Additional file 3: Table S3), the differentially expressed genes (blue) (see Additional file 3: Table S3) between V-0 and V-6 and the alternatively spliced genes (orange) (see Additional file 6: Table S6) between V-0 and V-6. B: Venn diagram comparing the expressed genes on V-0 and V-6 (see Additional file 3: Table S3), the differentially expressed genes (blue) (see Additional file 3: Table S3) between V-0 and V-6 and the alternatively spliced genes (orange) (see Additional file 6: Table S6) between V-0 and V-6. (TIFF 64679 kb) [file 12864_2018_4896_MOESM8_ESM.tiff]

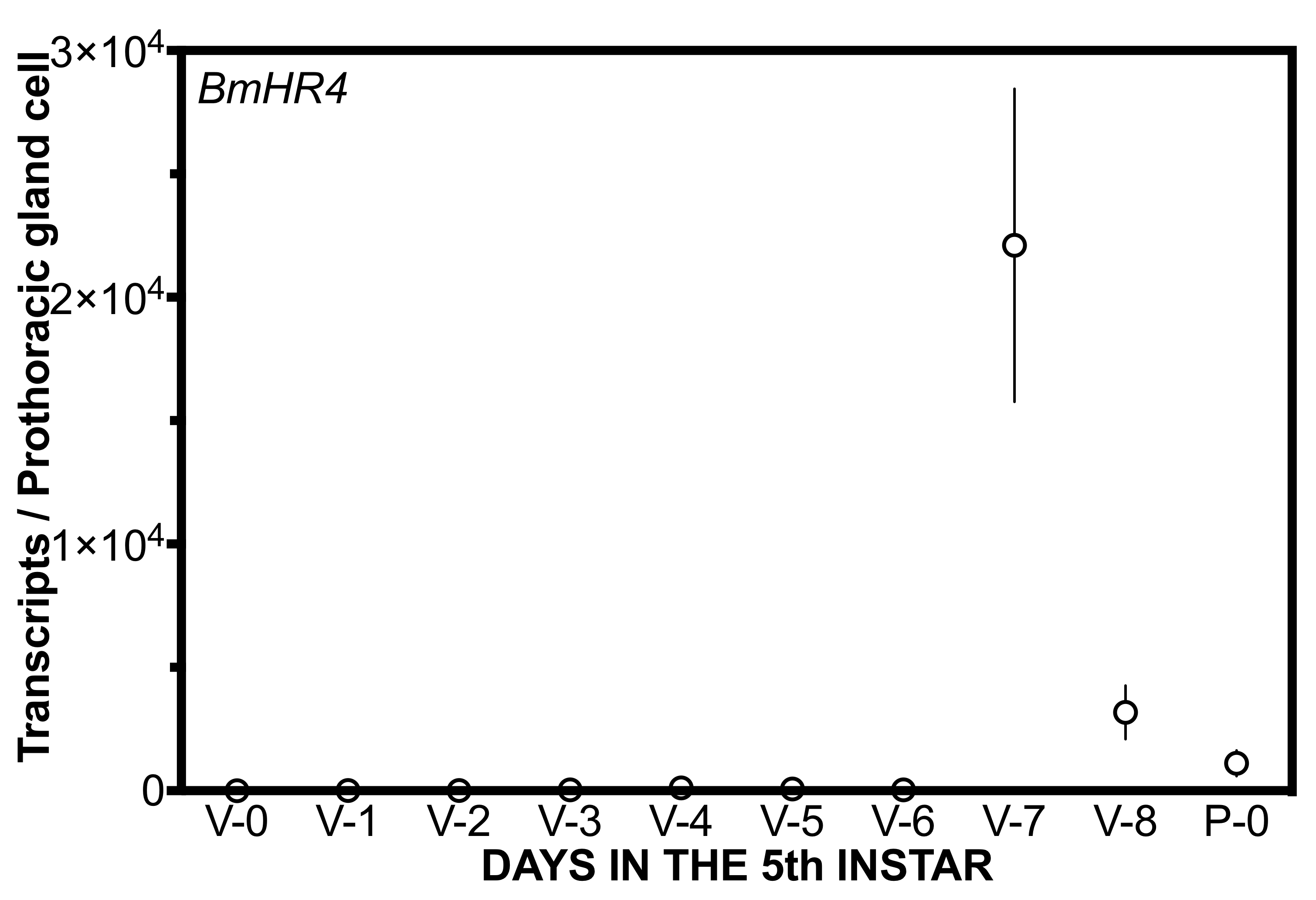

Supplement: Supplementary file 23 — Figure S6. Expression profile of BmHR4 during the final larval instar and the first day of the pupal stage of Bombyx mori. (TIFF 24374 kb) [file 12864_2018_4896_MOESM23_ESM.tiff]
